# Oxygen Delivery from Ethylcellulose/Calcium Peroxide Composite Films: Effects of Composition and Medium pH

**DOI:** 10.1021/acsomega.6c05828

**Published:** 2026-07-14

**Authors:** Camila Gruber Chiaregato, Denise Freitas Siqueira Petri

**Affiliations:** Instituto de Química, Universidade de São Paulo, 05508-900 São Paulo, Brazil

## Abstract

The delivery of O_2_ through the reaction of CaO_2_ with water is relevant for environmental and biomedical applications, which often operate across a broad pH range. However, the rapid reaction kinetics of CaO_2_ can cause an undesirable burst-release effect. Incorporating CaO_2_ into hydrophobic polymer matrices is a promising strategy to overcome this limitation. In this study, composite films based on ethylcellulose (EC), a hydrophobic cellulose ether, and varying CaO_2_ contents were developed and evaluated for oxygen release under different pH conditions. Surface roughness, hydrophobicity, and film thickness increased with increasing CaO_2_ content. Films containing the highest CaO_2_ loading (62 wt %) exhibited the greatest surface roughness (12 μm) and a contact angle close to 120°. This formulation most effectively controlled the reaction kinetics, reducing the reaction rate by up to 13-fold compared with pure CaO_2_, and produced the highest dissolved O_2_ concentrations in buffered media at pH 4.5 and 7.5. In contrast, in deionized water, rapid pH elevation caused by Ca­(OH)_2_ formation suppressed O_2_ generation. As a proof of concept, the biological effect of oxygen release from the composites was investigated using the root growth of *Allium cepa*. The results showed that both buffered conditions and excessive CaO_2_ content inhibited root development. However, films containing 62 wt % CaO_2_ in deionized water enhanced root length, demonstrating that the beneficial effects of oxygen release depend strongly on both the medium characteristics and CaO_2_ concentration.

## Graphical abstract

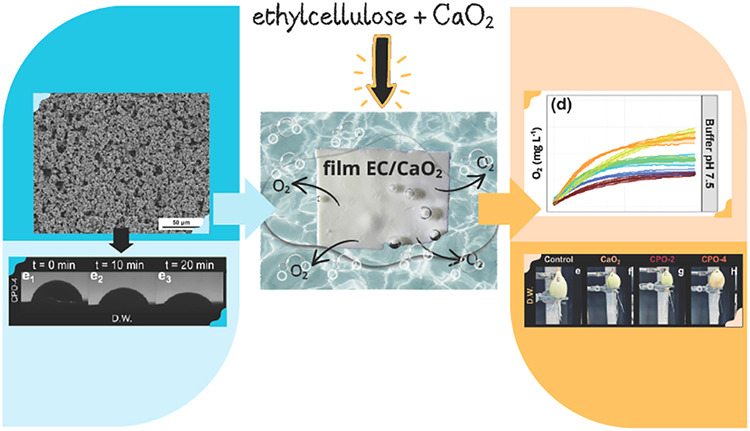

## 1 Introduction

Oxygen (O_2_) is essential for aerobic metabolism, and insufficient levels can cause hypoxia and cell death.^1^ Since factors such as pollutants, temperature, pressure, and pH can reduce the level of O_2_, strategies to restore oxygen levels are often needed. One approach is the use of peroxide-based oxygen sources, which release O_2_ upon contact with water without requiring a continuous energy input.

Several peroxides can be used as O_2_ suppliers, including hydrogen peroxide (H_2_O_2_), sodium percarbonate (Na_2_CO_3_ *·*1.5H_2_O_2_), magnesium peroxide (MgO_2_), and calcium peroxide (CaO_2_). Solid peroxides are advantageous due to easier handling, transport, and storage. Among them, CaO_2_ is widely used because of its low cost, high purity, and relatively high theoretical reactive oxygen content (22.22 wt %).^2^ The reaction of CaO_2_ with water can be represented by

1 $$2{\mathrm{CaO}}_{2}+2H_{2}O\to 2\mathrm{Ca}{\left(\mathrm{OH}\right)}_{2}+O_{2}$$

2 $${\mathrm{CaO}}_{2}+2H_{2}O\to \mathrm{Ca}{\left(\mathrm{OH}\right)}_{2}+H_{2}O_{2}$$

3 $$H_{2}O_{2}\to H_{2}O+1/2O_{2}$$

The reaction of CaO_2_ with water can follow two different pathways: (i) direct formation of O_2_ (eq 1), or (ii) initial production of H_2_O_2_ (eq 2), which subsequently decomposes into O_2_ (eq 3). Both pathways produce Ca­(OH)_2_, which raises the medium pH in unbuffered systems. These reactions are fast and exothermic processes,^3^ and the reaction kinetics are influenced by the initial calcium peroxide concentration, oxygen conditions (hypoxia vs normoxia), and the presence of catalase.^4^ Under hypoxic conditions, the reaction rate tended to be higher, while increased CaO_2_ concentration and catalase enhanced oxygen yield.^4^

Peroxide release kinetics are strongly influenced by pH and buffer composition. Citrate buffer enhanced O_2_ release from less soluble peroxides, while phosphate favored CaO_2_ and Na_2_CO_3_ *·*1.5H_2_O_2_. Carbonate buffer at alkaline pH further increased O_2_ release, likely due to the catalytic effect of carbonate ions.^5^ A stabilized amorphous calcium carbonate nanocoating on CaO_2_ was shown to suppress its reaction with water, with oxygen release regulated by adjusting NaH_2_PO_4_ and NaHCO_3_ concentrations at different pH conditions.^6^ These findings demonstrate that the reactions between CaO_2_ and water are complex and influenced by multiple factors.

The use of CaO_2_ as an oxygen supplier has been investigated in environmental remediation and medical applications.^2^ In both fields, balancing oxygen release and toxicity is critical. To minimize pH-related side effects and prolong oxygen release, coating CaO_2_ with different materials has become a common strategy. Among these materials, polymers are widely used due to their versatility, ease of modification, and broad range of properties. Several polymer-based systems have been developed for CaO_2_ encapsulation. For example, CaO_2_ has been incorporated into hydrophobic polycaprolactone (PCL) particles within fluorinated chitosan matrices,^4^ thiolated gelatin cryogels,^7^ chitosan/poloxamer/hyaluronic acid systems,^8^ poly­(l-lactide-*co*-glycolide) (PLGA) composite films, and electrosprayed collagen–whey protein films.^9^

Cellulose-based materials are promising platforms for CaO_2_ incorporation in environmental and biomedical applications because of their biocompatibility and biodegradability.^10,11^ For example, hydroxypropyl methylcellulose (HPMC)/CaO_2_ cryogels showed tunable oxygen release depending on the degree of substitution and the molar substitution of HPMC, which affected swelling behavior and water interaction.^3^ Lignocellulose fiber/CaO_2_ xerogels also provided sustained O_2_ release, promoting root growth of *A. cepa*.^12^ Among hydrophobic cellulose derivatives, there are no reports on the use of ethylcellulose (EC) as a matrix to retard the reaction between CaO_2_ and water. EC is a hydrophobic, film-forming polymer with tunable properties and a comparatively low environmental impact upon disposal.^13–17^ Moreover, EC is readily soluble in ethanol, which acts as an inert medium for CaO_2_.

In this study, EC/CaO_2_ composite films with different CaO_2_ contents were developed to test the hypothesis that the hydrophobic character of EC might serve as a kinetic rate controller for the reaction between CaO_2_ and water and, consequently, for the O_2_ delivery. Considering that environmental and biomedical applications may occur across a wide pH range, the stability of the EC films and the kinetics of O_2_ release were investigated as a function of pH. As a proof of concept, the efficiency of EC/CaO_2_ composites as O_2_ suppliers was evaluated for the root growth of *A. cepa* in different pH media and initial concentrations of CaO_2_ (pure and composite). The *A. cepa* root growth test is a well-established method in environmental studies due to its low cost, ease of handling, sensitive response, and good correlation with other test systems.^18,19^

## 2 Materials and Methods

### 2.1 Materials

Ethylcellulose (EC, 48% ethoxylated, viscosity of 100 cP in a mixture of toluene and ethanol 80:20, Sigma-Aldrich—247499), calcium peroxide (CaO_2_, 90% purity, Sigma-Aldrich—466271), ethanol (99% purity, LabSynth), tris­(hydroxymethyl) aminomethane (Tris base, 99.8% purity, LabSynth), hydrochloric acid (HCl, 37%, LabSynth), sodium hydroxide (NaOH, 97% purity, LabSynth), acetic acid (CH_3_CO_2_H, 99.7% purity, LabSynth), sodium acetate (CH_3_COONa, 99% purity, LabSynth), and deuterated chloroform (CDCl_3_, 99.8% purity, Sigma-Aldrich) were used as received. All the solutions were prepared using deionized water (D.W., pH 5.5, conductivity of 4.85 μS cm).

### 2.2 Preparation of EC and EC/CaO_2_ Films

Figure 1 depicts the experimental steps to prepare EC and EC/CaO_2_ films. Initially, EC was dissolved in ethanol at 5 wt % under magnetic stirring, at 24 *±* 1 °C, for 24 h. For the formulations containing CaO_2_, the nominal proportions of EC:CaO_2_ (w/w) were 1:0.5, 1:1, 1:1.5, 1:2, and 1:4, see Table 1. These ratios were established based on the sensor limitations for O_2_ quantification, which has a maximum detection capacity of 20 mg L^*–*1^ (0.62 mmol L^*–*1^). The CaO_2_ loading calculations were adjusted using the effective purity factor, determined by thermogravimetric analyses of CaO_2_. The maximum concentration of O_2_ at 1 atm ranges between 1.18 and 1.25 mmol L^*–*1^ at 25 °C; however, considering the composition of air and the O_2_ partial pressure of 0.21 atm, the solubility decreases to 0.256 mmol L^*–*1^ (8.1 mg L^*–*1^).^20^ To effectively disperse the CaO_2_ particles in the EC solution, the beaker containing the system was placed in an ice bath, and the ultrasonic homogenizer (Hielsher, Germany) was set at 60% of maximum power for 30 s, followed by magnetic stirring at 200 rpm for 10 min to ensure complete homogenization, which was visually observed. For film formation, 2 g of the dispersion was poured into silicone molds (5 cm in diameter) and oven-dried at 60 °C until the complete evaporation of ethanol. Drying time varied by composition, ranging from 30 min to 1.5 h. After drying, 2.5 cm diameter samples were cut from the films, discarding the visually thicker edges (as shown in Figure S1). Then, all films were stored in a desiccator until characterization or further use. After storing the films in the desiccator for over one year, no mass loss or changes in the film appearance could be observed.

**Figure 1:**
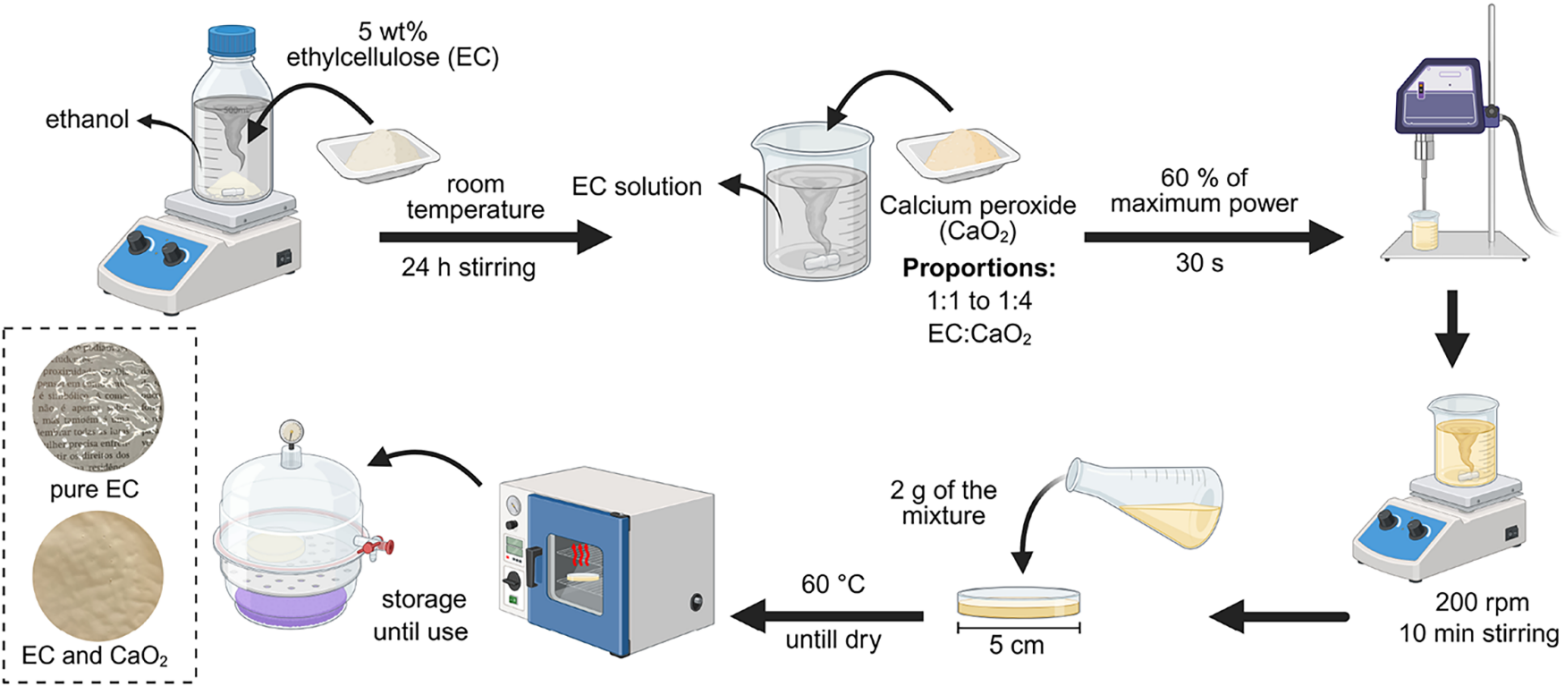
Experimental methodology diagram. Created in BioRender. Chiaregato, C. (2026). https://BioRender.com/adg3o32.

**Table 1:** Composite Formulations with Their Respective Codes, Nominal Proportions Between EC Polymer and CaO_2._ and the Corresponding Contents^a^

sample code	proportion	EC (wt %)	CaO_2_ (wt %)	density (g/cm^3^)
CPO–0	1:0	100	0	0.7527 ± 0.0520
CPO–0.5	1:0.5	66.67	33.33	1.0016 ± 0.0692
CPO–1	1:1	50	50	0.9109 ± 0.0575
CPO–1.5	1:1.5	40	60	1.0347 ± 0.1197
CPO–2	1:2	33.33	66.67	1.1260 ± 0.0558
CPO–4	1:4	20	80	0.8733 ± 0.0376

a Mean bulk density values (*n* = 2) and standard deviations were determined for the CPO films.

### 2.3 Characterization of EC and EC/CaO_2_ Films

^1^H nuclear magnetic resonance (NMR) of the EC polymer analysis was performed following the methodology described by Hasani and Westman.^21^ The spectrum was recorded at 25 °C; the solvent used was CDCl_3_ (BRUKER 500 MHz). The acquisition parameters were spectral width of 20 ppm, acquisition time of 3 s, and 32 scans. The chemical shifts were reported as ppm values using the zg30 pulse sequence. Baseline correction, Fourier transformation, and integration were performed using Bruker Topspin 4.4.1 software. The degree of substitution (DS) was calculated based on values from the integrated peaks, using eq 4 described by Heinze et al.,^15^ where I_H,ethyl_ is the sum of integrated areas from the signals from −CH_2_–CH_3_ ethyl group, and I_H,AGU_ is the sum of integrated areas from the signals from cellulose glucose monomer:

4 $$\mathrm{DS}=3-\frac{\left(7\times I_{H,\mathrm{ethyl}}\right)}{\left(3\times I_{H,\mathrm{AGU}}\right)}$$

Differential scanning calorimetry (DSC) analysis, following the methodology described by Lai et al.^22^ with modifications, of the EC polymer was done using an inert atmosphere of N_2_ with a flow rate of 50 mL min^*–*1^, with the pan open (punched on the top) or sealed (DSC Q10, TA Instruments). Analysis program: isotherm at 0 °C during 5 min; 1st heating with the heat rate of 5 °C min^*–*1^ and *T*_max_ of 250 °C; 1st cooling with the cooling rate of 10 °C min^*–*1^ and *T*_min_ of 0 °C; 2nd heating with the heat rate of 20 °C min^*–*1^ and T_max_ of 270 °C.

The thermogravimetric analyses (TGA) were done using an inert atmosphere of N_2_ with a flow rate of 60 mL min^*–*1^ and the heating rate of 10 °C min^*–*1^ between 20 and 900 °C (TGA, Netzsch, STA 449 F3 Jupiter, Q500 TA Instruments). The analysis was performed on formulations CPO–2 and CPO–4 (before and after the kinetics reaction in buffer Tris-HCl, pH 7.5), pure EC, and CaO_2_. To calculate the CaO_2_ content or purity, the following equations were used

5 $$2{\mathrm{CaO}}_{2}\to 2\mathrm{CaO}+O_{2}$$

6 $$2\mathrm{Ca}O_{2}\left(\%\right)=\frac{\Delta m}{m_{0}}\times 4.505\times 100$$

where Δ*m* is the mass loss of the sample (stage of weight loss II, Table S2), *m*_0_ is the initial mass of the sample, and 4.505 is the stoichiometric factor of the reaction (eq 5).^23^

Fourier transform infrared (FTIR) spectroscopy analyses were performed using the attenuated total reflectance (ATR) mode and a ZnSe/diamond crystal, accumulation of 64 scans, and resolution of 4 cm^*–*1^, in the range of 600 to 4000 cm^*–*1^ (Frontier, PerkinElmer). The analysis was performed on all of the EC/CaO_2_ formulations, pure EC, and CaO_2_.

The thickness measurements were carried out using 10 independent films of the same composition and a digital pachymeter Digimess. The film volume was estimated as the product of the film area and the average thickness. The bulk density of the films was estimated for duplicates by dividing the mass by the corresponding estimated volume. Scanning electron microscopy (SEM) analyses were performed in high-vacuum mode using the secondary electron detector (SE) at 10 kV (JEOL Neoscope JCM-5000). All of the samples were coated with a 20 nm Au layer. For the analysis of the samples after the kinetics reaction, the films were first oven-dried at 60 °C for 2 h. Energy dispersive X-ray (EDS) mapping analyses were performed with a Thermo Noran System Six equipped with a Si­(Li) detector beam at 20 kV, spot size of 6, working distance of 20 μm.

Surface topography was analyzed using Coherence Scanning Interferometry (CSI) (Taylor Robson Inc.). Measurements were conducted using a 50x objective lens. For each sample, four different areas of 300 μm were scanned, and the average of the surface area roughness (S_a_) was calculated after the application of a Gaussian filter using the software TalyMap Gold 6.2.6613.0 (Taylor Robson Inc.). The CPO–0 film was coated with a 20 nm Au-o layer to reduce laser reflection.

The advancing contact angle measurements (SEO Phoenix – I, Korea) were done using the sessile drop method using 8 μL of: (i) D.W. (pH 5.5), (ii) 100 mM Tris base–HCl buffer (pH 7.5), and (iii) 100 mM acetate–acetic acid buffer (pH 4.5). For the receding contact angle measurements, half of the droplet volume was removed. Measurements were performed and presented as the average and standard deviation of 6 replicates for each medium and material. The photographs were taken right after the droplet was deposited on the surface. The contact angle was determined using the ″Drop analysis LB ADSA″ plugin for ImageJ software, as described and developed by Stalder et al.^24^ To evaluate the stability of the CPO–0 films in different media, the films (2.5 cm in diameter) were immersed for 24 h in separate volumes (20 mL) of 100 mM acetate–acetic acid buffer, pH 4.5; D.W., pH 5.5; 100 mM TRIS base–HCl buffer, pH 7.5; and 2 M NaOH solution, pH 11. After this period, they were removed from the media, air-dried at room temperature conditions (24 *±* 1 °C) for 24 h, and analyzed using SEM analysis

### 2.4 Release Kinetics of O_2_

The kinetics of O_2_ release from the reaction between CaO_2_ and water were investigated in (i) D.W. (initial pH of 5.5 and final pH of 11); (ii) 100 mM Tris base–HCl buffer (pH 7.5); and (iii) 100 mM acetate–acetic acid buffer (pH 4.5). These pH conditions represent an unbuffered system, buffered medium close to the physiological pH, and an acid-buffered medium (found in the environment due to acid mine drainage or acid rain^25^). The test specimens were cut into a circular shape with a diameter of 2.5 cm to ensure that the film interacted with the medium equally on all sides. First, a volume of 50 mL was purged with N_2_ gas to decrease the concentration of dissolved O_2_ to less than 1.2 mg L^*–*1^ (0.042 mmol L^*–*1^) and to mimic hypoxic conditions. A selective O_2_ sensor (Lutron WA-2015) was used to measure the dissolved oxygen (DO), while the temperature was maintained at 20.0 *±* 0.1 °C using a thermostatic bath (Thermo Scientific, Accel 250), as shown in the Supporting Information S3. The glass vial was sealed with Parafilm tape. The system was magnetically stirred for 4 h to ensure equilibrium, and the medium pH was measured after each experiment. CaO_2_ release kinetics are reported as the average of three replicates. For comparison with CPO–2 and CPO–4 films, pure CaO_2_ release kinetics were also measured under identical conditions using equivalent CaO_2_ concentrations of 0.936 and 1.86 mg L^*–*1^, respectively.

To calculate the CaO_2_ reaction rate, only the initial stage of the reaction was considered, as this region exhibited the greatest linearity and the particle surface area could be assumed approximately constant. Therefore, the initial reaction rate (*V*_0_) was determined from the slope of this initial region, which was based on the rate of O_2_ production. The *V*_0_ was calculated separately for each replicate by applying a linear regression during the first 30 min of reaction time for the films and first 15 min for pure CaO_2_ kinetics. The decision to use different time intervals was based on reaction speed: for pure CaO_2_, the reaction was faster, and after 15 min, the curve began to deviate from linearity. The maximum concentration of O_2_ released and the time necessary to achieve it were determined for each replicate. All values were presented as the average and the corresponding standard deviation. The ratio between the *V*_0_ values for pure CaO_2_ and films in different media was calculated using eq 7, where V_0,CaO2_ is the initial velocity for pure CaO_2_ and *V*_0,film_ is the initial velocity for CPO–2 or CPO–4 film.

7 $$r=\frac{V_{0,\mathrm{CaO}2}}{V_{0,\mathrm{film}}}$$

### 2.5 Root Growth of *Allium cepa*

To evaluate the performance of the composites as O_2_ suppliers, root growth of *A. cepa* considering: (i) medium pH, (ii) O_2_ supply from films versus pure CaO_2_, and (iii) the initial mass of the film or pure CaO_2_. The onions were purchased from a local market and selected only based on visual inspection, considering similar size and apparent health. Despite the visual limitations, only onions that had similar features were chosen. The effect of pH on onion root growth was investigated in D.W. (initial pH 5.5), a 100 mM acetate–acetic acid buffer (initial pH 4.5), and a 100 mM Tris base–HCl buffer (initial pH 7.5). Each onion was placed in a separate 50 mL vial containing the corresponding medium. To evaluate the impact of oxygen supply on root growth, four treatments were tested: (i) a control group (containing only the medium); (ii) in the presence of pure CaO_2_ at a concentration of 1.86 mg L^*–*1^; and in the presence of either (iii) CPO–2 films or (iv) CPO–4 films. The vials were positioned according to the natural light entering through the window, either in a predetermined arrangement based on treatment (Experiment 1) or in a random arrangement (Experiment 2), as illustrated in Figure 2. In Experiment 3, the initial mass was increased to 5 times that used in the previous experiment to assess the influence of initial mass.

**Figure 2:**
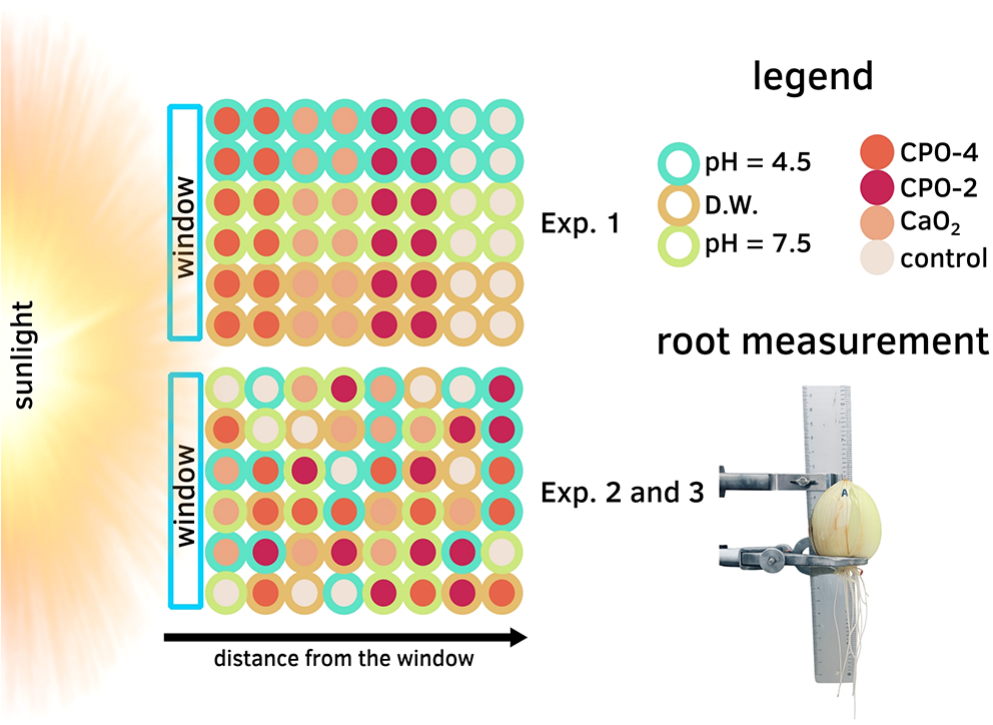
Illustration showing the distribution of vials in each experiment according to treatment and medium.

The onion roots were photographed every 3 days, and the images were analyzed using ImageJ. The pH of each medium was measured every 2 days to track the progression of the CaO_2_ reaction. The experiment was conducted from June to August (winter season in Brazil), with temperatures ranging from 13 to 22 °C.

The results for *A. cepa* root growth are presented as the average of four independent replicates per condition. The root growth measurements reflect the total length of all roots per onion rather than an average length. This approach was chosen because numerous variables can influence root growth beyond the O_2_ supply alone. As a result, oxygen supply benefits may appear in different forms, such as thicker roots, even if fewer in quantity. Given the complexity of these factors, the focus was on the total length of the roots. Additionally, the average values are influenced by extreme values (outliers). The complete statistical analysis of the root growth measurements is available in Supporting Information S4.

### 2.6 Software and Statistical Data Treatment

All images and graphs were assembled and structured using Inkscape (v. 1.4.2) and R software (version 4.5.2) with Colorblind-Friendly Color Maps^26^ and ImageJ software (version 1.53t).^27^ Generalized linear mixed models (GLMMs) were used to evaluate onion root growth across treatments. The data were treated as a continuous positive variable and modeled with a Tweedie distribution and a log link. To compare Experiments 1 and 2, an additive model was used; to compare Experiments 2 and 3, an interaction model was used. The number of replicates for each experiment is specified in the caption of the corresponding graph.

## 3 Results and Discussion

### 3.1 Determination of DS and Thermal Properties of EC

Initially, the EC degree of substitution (DS) was determined by ^1^H MNR analysis, and DSC was used to assess its thermal properties. Figure S5 shows the ^1^H MNR spectrum, where proton signals from δ = 4.33, 3.94, 3.72, 3.53, 3.34, 3.21 ppm correspond to the cellulose glucose monomer, and the signals from δ = 1.72 ppm; CH_3_, 1.19 ppm correspond to the −CH_2_–CH_3_ ethyl group.^28^ The DS was calculated (eq 4) as 0.91. The signal at 7.26 ppm corresponded to the CDCl_3_ solvent used to dissolve EC.^29^

Figure S6 shows the DSC curves obtained for cooling and 2nd heating curve. Both methodologies (sealed and pin-holed pans) showed a first-order melting event (*T*_fusion_ = 185–195 °C), an exothermic crystallization event (*T*_crys._ = 227–236 °C), and a glass transition (*T*_g_ = 111–132 °C). The values obtained for each event (Table S7) are consistent with the reported values in the literature.^13,30^

### 3.2 Determination of EC/CaO_2_ Film Composition and Structural Characterization

TGA analysis (Figure S8) was used to determine the CaO_2_ purity and the composition of the CPO–2 and CPO–4 films, selected as representative samples with the highest CaO_2_ contents. The complete weight loss stages (Δ_mass_) and the corresponding *T*_max_ values are provided in Table S1. Pure CaO_2_ showed a calculated purity (eq 6) of 90%, consistent with the supplier’s reported range of 70–90% purity.

The experimental CaO_2_ contents of CPO–2 and CPO–4 were (57 *±* 8.5) wt % and (62 *±* 4.2) wt %, respectively, lower than the theoretical values of 67 and 80%. This discrepancy was likely caused by a ″coffee-ring″ effect during drying,^31^ where solids migrated toward the film edges, reducing CaO_2_ concentration at the center of the sample. Since all analyses were performed on 2.5 cm disks cut from the film center, the measured values were lower than expected (Figure S1). Stage I of weight loss was assigned to EC degradation (Table S2). The addition of CaO_2_ to the EC resulted in a loss of thermal stability, as *T*_max_ decreased from 352 °C (pure EC) to values ranging between 325 and 332 °C (CPO–4 and CPO–2). This suggested that the interactions between EC and CaO_2_ were weaker than those among EC chains, implying that CaO_2_ was not chemically bonded to the EC chains but instead was physically mixed (anchored).

Figure S9 shows the FTIR spectra of pure EC, pure CaO_2_, and the EC/CaO_2_ films. For pure EC, the spectra presented the typical vibrational bands.^30,32,33^ The broad band at 3480 cm^*–*1^ corresponds to the O–H asymmetric stretching vibration, ν_*as*_(−OH), of glucosidic rings with hydroxy groups not replaced by −CH_2_CH_3_ during etherification. The bands at 2973 and 2871 cm^*–*1^ correspond to the C–H asymmetric stretching ν_*as*_(CH_3_) group. The band at 2930 cm^*–*1^ was attributed to asymmetric stretching ν_*as*_(−CH_2_) group. The bands at 1375 and 1050 cm^*–*1^ were assigned to symmetric bending vibration δ_*s*_(−CH_3_) group and symmetric stretching vibration ν_*s*_(C–O–C) of the glycosidic linkage, respectively.^29^ The spectra obtained for pure CaO_2_ presented bands at 856 cm^*–*1^ and 875 cm^*–*1^ assigned to the stretching vibration δ (O–O) oxygen bridge.^34^ The band at 1420 cm^*–*1^ corresponds to the bending vibration ν­(O–Ca–O*)*.^25,37^ The small band at 1457 cm^–1^ was attributed to the vibration of CO_3_^2–^.^35,36^ In general, all the films showed the characteristic bands of both EC and CaO_2_. Comparing the spectra of pure EC or CaO_2_ with those of the composite films, it was not possible to observe any band displacement or disappearance, indicating that no new chemical bonds were formed after the mixture, corroborating TGA analysis.

### 3.3 Characterization of EC and EC/CaO_2_ Film Surface

Figure 3 shows digital photographs, SEM images, and mean thickness values obtained for EC and composite films. CPO–0 films were transparent, while the EC/CaO_2_ films exhibited a yellowish color, which became more intense as the CaO_2_ content increased. The mean film thickness increased from 75 *±* 14 μm to 285 *±* 23 μm, following the increase in CaO_2_ content. CPO–0 (Figure 3a) exhibited a flat, uniform morphology, whereas the surfaces of the EC/CaO_2_ films became thicker, rougher, and more irregular as the CaO_2_ content increased (Figure 3b–f). For CPO–4 (Figure 3f), the high CaO_2_ content led to the formation of voids, creating a porous structure as a consequence of the packing or agglomeration of CaO_2_ particles. Even though CPO–2 (57 wt %) and CPO–4 (62 wt.%) had similar CaO_2_ contents, the resulting morphologies were markedly different.

**Figure 3:**
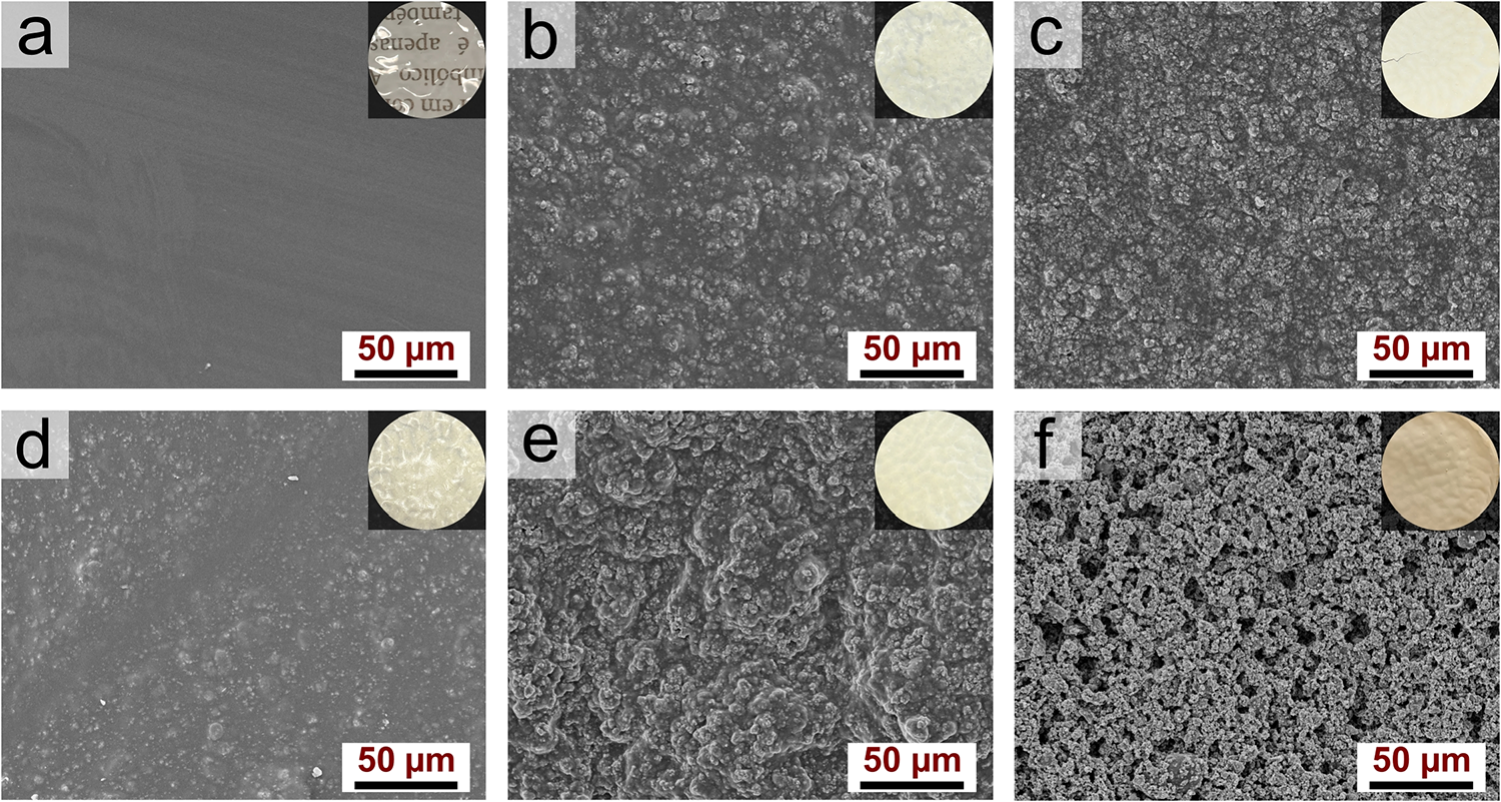
SEM images of EC and composites, along with their photographs (top right corner) and corresponding average thickness values (*n* = 10): (a) EC, 75 *±* 14 μm; (b) CPO–1, 103 *±* 18 μm; (c) CPO–2, 161 *±* 16 μm; (d) CPO–0.5, 101 *±* 19 μm; (e) CPO–1.5, 147 *±* 13 μm; and (f) CPO–4, 285 *±* *23* μm.

The EDX mapping for Ca (Figure 4) shows the distribution of Ca ions on the surface of the composite films. Although EDX is less reliable for detailed analysis of light elements such as C and O, no Ca was detected on the CPO–0 film (Figure 4a), as expected. For CPO–2 (Figure 4b), the Ca distribution was concentrated in specific areas, forming strip-like patterns, which corroborates with the uneven distribution of solid particles during the drying process. This heterogeneous distribution of Ca might explain the differences in CaO_2_ content between nominal and experimental values. For CPO–4 (Figure 4c), the Ca distribution was more homogeneous across the surface.

**Figure 4:**
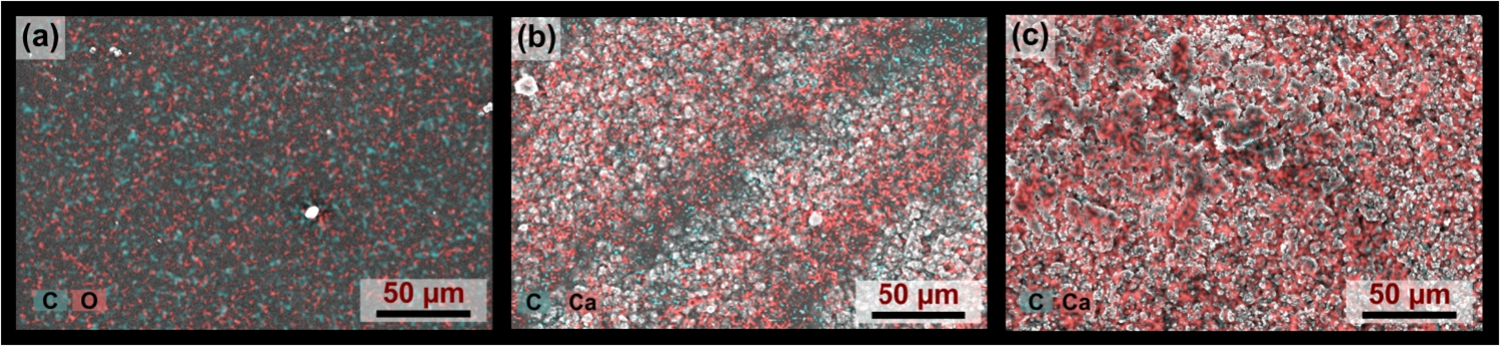
SEM images of the surface of EC and EC/CaO_2_ films with energy dispersive X-ray (EDX) mapping for (a) C (green) and O (red) in EC; (b) C (green) and Ca (red) in CPO–2; and (c) C (green) and Ca (red) in CPO–4.

To quantify surface roughness (*S*_a_), all films were analyzed using profilometry (CCI). The *S*_a_ value is the arithmetic average of the absolute values of the surface height deviations measured from the mean height. Figure S10 shows typical mapping images over 300 μm × 300 μm areas for EC and EC/CaO_2_ films.

Figure 5 shows *S*_a_ values ranging from 4 to 12 μm across different compositions. Compared with pure EC films, all composites exhibited higher *S*_a_ values, indicating increased surface roughness with the addition of CaO_2_. The increase in surface roughness might promote the formation of micro- and nanochannels that allow water to penetrate and contact the particles. Among the composites, CPO–4 showed the highest *S*_a_ value, whereas CPO–0.5 to CPO–1.5 had similar *S*_a_ values. CPO–2 showed two groups of *S*_a_ values, as indicated by the jitter distribution in the boxplot (Figure 5), which affected the mean value. SEM images (Figure 3b–f) show agglomerated CaO_2_ particles on the surface, corroborating the S_a_ results.

**Figure 5:**
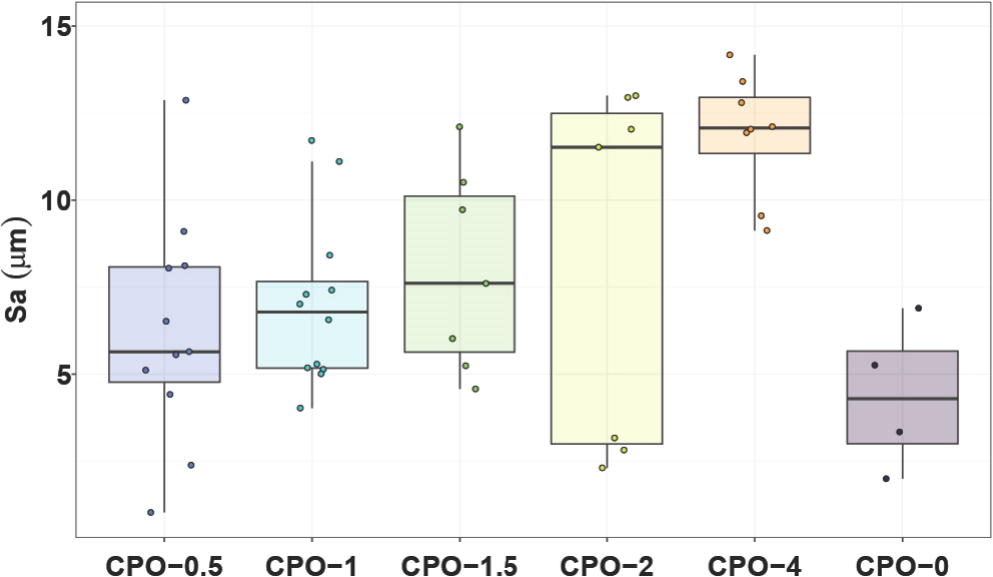
Boxplot of surface (area) roughness (*S*_a_) for EC and EC/CaO_2_ (*n* = 6).

One should note that in general, the film thickness increased with CaO_2_ content relative to CPO–O films, and within the same composition, a thickness gradient from the center to the edge of the film was evidenced by the surface profilometry (Figure S11). Both effects could be attributed to the drying process and packing of CaO_2_ particles within the EC matrix, leading to a more voluminous structure. However, Table 1 shows that the bulk density values did not proportionally increase with the CaO_2_ content in the formulation. This behavior is likely associated with the presence of air trapped within the material, the porous structure of the films (Figure 3), and possible overestimation of the thickness due to surface roughness (Figure 5) and thickness heterogeneity.

So far, it has been observed that the film’s surface roughness and morphology change with the CaO_2_ content. These surface characteristics can affect how the medium interacts with the film, which in turn influences the kinetics of O_2_ release. To explore this interaction further, we measured the films’ contact angles using the sessile drop method, as shown in Figure 6. The contact angle was measured at the solid–liquid interface across all compositions and media used in the kinetics study. While surface roughness might lead to overestimating contact angles due to air entrapment, it still offers valuable insights into the surface properties of the films and their interaction with different media.

**Figure 6:**
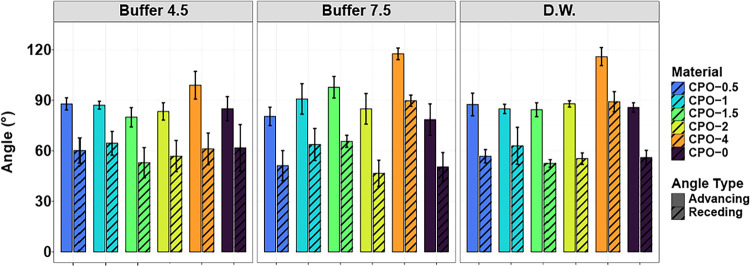
Contact angle measurements using droplets (8 μL) of acetic acid-acetate buffer, pH 4.5; tris-HCl buffer, pH 7.5; and D.W., pH 5.5 on EC and composite films (*n* = 6).

The wetting properties were assessed using advancing (θ_*a*_) and receding (θ_*r*_) contact angles in the sessile drop method. Figure 6 shows that the θ_*a*_ values ranged from 80 to 120°, revealing the hydrophobic nature of EC and the composite films. The highest contact angles were observed for CPO–4 across all aqueous media. CPO–4 had the highest *S*_a_ value (Figure 5) and irregular surface (Figure 3), which favored air entrapment and increased hydrophobicity (Cassie–Baxter regime). In addition, the surface of a rough solid is energetically heterogeneous.^37^ In all cases, the contact angle hysteresis Δθ (Δθ = θ_*a*_ – θ_*r*_) was higher than 10°, which might be due to the surface heterogeneity and roughness,^38^ consistent with the observations in Figures 3 and 5.

Figure S12 illustrates the changes in contact angles at the solid–liquid interface between the medium (D.W. or buffer at pH 7.5) and the surface of the EC/CaO_2_ composites. The droplets were photographed at 0, 10, and 20 min after droplet deposition. The observed decrease in the contact angle over time may be attributed to surface changes caused by the reaction between CaO and water and to changes in droplet composition due to the release of Ca­(OH)_2_, H_2_O_2_, and O_2_ into the medium.

### 3.4 Kinetics of Oxygen Release

Before evaluating the O_2_ release kinetics, the stability of the EC films was evaluated in four media: (a) acetic acid-acetate buffer, pH 4.5; (b) D.W., pH 5.5; (c) Tris–HCl buffer, pH 7.5; (d) NaOH solution, pH 11. SEM images (Figure S13) confirmed that the films remained intact after 24 h of immersion in all media, with no cracks or holes. This indicates that the polymer matrix remained stable and retained its structural integrity. Therefore, the observed O_2_ release behavior can be attributed to interactions among the polymer, CaO_2_, and the medium, rather than to film instability.

In this work, O_2_ released into the aqueous medium was determined as a function of time. The released O_2_ in the medium over 4 h is probably controlled by water diffusion (eq 1) rather than by H_2_O_2_ decomposition (eq 3). The decomposition of H_2_O_2_ at pH 7.4 and 20 °C is relatively slow and may require more than 48 h to reach completion, as reported by Pędziwiatr et al.^39^ and Ghaffari-Bohlouli et al.^4^

The O_2_ release profiles (Figure 7) reflect both the reaction between CaO_2_ and water and atmospheric O_2_ diffusion.^12^ Because O_2_ permeated through the Parafilm, atmospheric O_2_ gradually diffused into the medium. To account for this effect, a control experiment without material (“diffusion”) was performed. The values of O_2_ concentration represent the equilibrium between O_2_ released from the reaction between CaO_2_ and water, and O_2_ diffused from the atmosphere. As atmospheric diffusion was part of this equilibrium, its contribution was not subtracted from the total O_2_ concentration values.^12^

**Figure 7:**
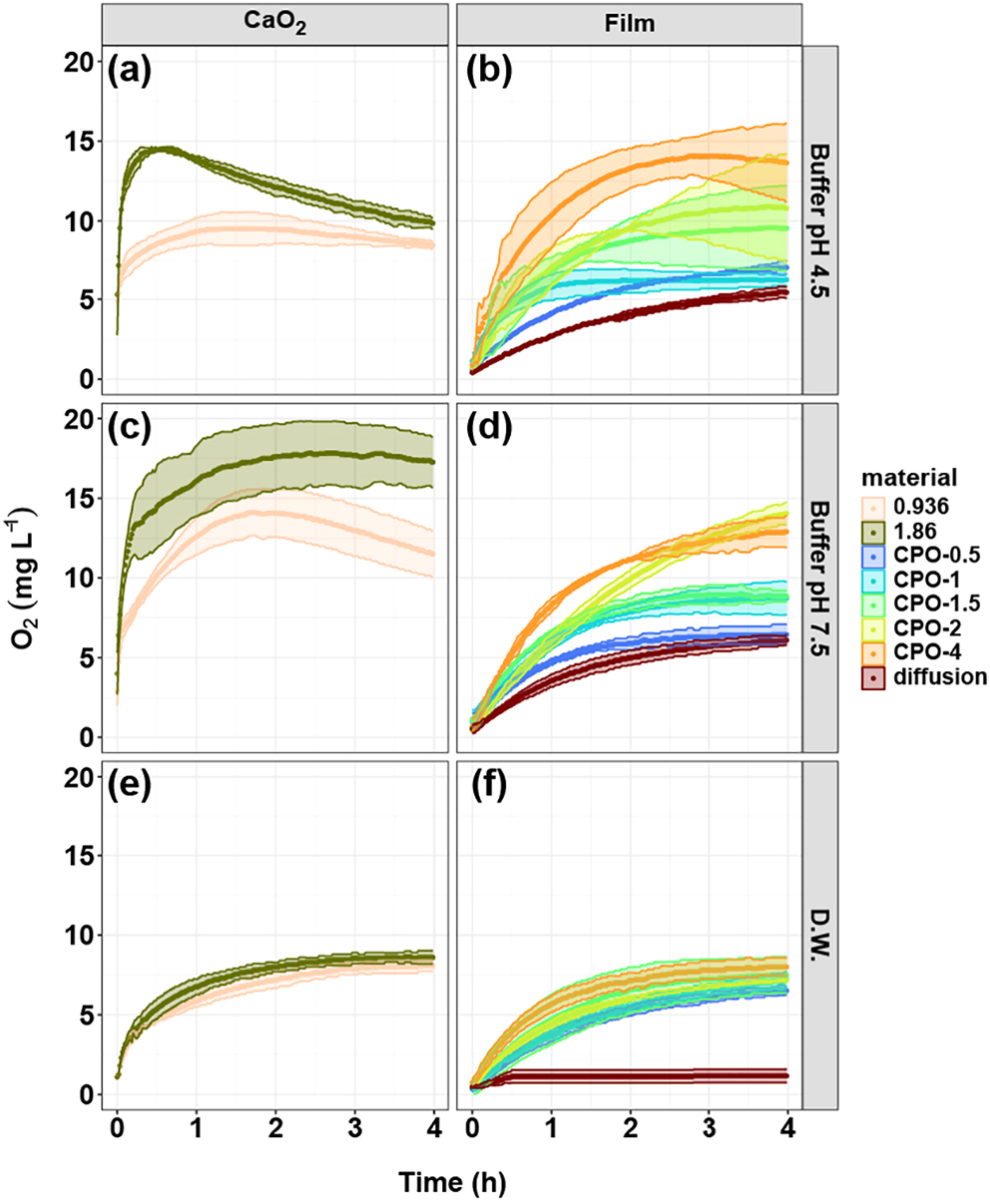
Kinetics release of O_2_ at 20.0 *±* 0.1 °C in different reaction mediums for pure CaO_2_: (a) buffer pH 4.5; (c) buffer pH 7.5; (e) D.W.; and EC/CaO_2_ films: (b) buffer pH 4.5; (d) buffer pH 7.5; (f) D.W. Diffusion = kinetics without any material that could generate O_2_. The mean values (thick lines) and the corresponding deviations (thin lines) were obtained for at least three independent replicates.

The kinetics followed three typical stages: (i) a rapid initial phase, where the linear behavior allows for the determination of initial velocity V_0_; (ii) an intermediate, slower phase, due to the heterogeneous reaction and changing particle surface; and (iii) an equilibrium stage, when O_2_ concentration stabilizes. Burst release phenomenon was only observed for CaO_2_ microparticles in buffered systems. This effect intensified at a concentration of 1.86 mg L^*–*1^, likely due to an increase in surface area, which reflected in a higher reaction rate (Table 2), as also observed by Chen et al.^35^ and Wang et al.^40^ No burst release has been observed for EC/CaO_2_ films, indicating that the encapsulation of CaO_2_ within the polymer matrix effectively mitigated this effect. Previous studies have reported that decreasing the polymer ratio generally increases burst release.^41^ However, in the present study, the fact that CPO–4 contains only 38 wt % EC relative to the CaO_2_ microparticles highlights that even a relatively small amount of polymer was sufficient to slow the reaction and prevent burst release, probably because this composition presented the highest contact angle values (Figure 6).

**Table 2:** Mean Values of Initial Reaction Velocity (*V*_0_,mg L^–1^ h^–1^) and the Corresponding SD Across Different Media, and *r* Values (Eq 7)

sample	buffer pH 4.5			buffer pH 7.5			D.W.		
	*V*_0_	SD	*r*	*V*_0_	SD	*r*	*V*_0_	SD	*r*
CaO_2_ 0.936	1.92	0.15		1.86	0.16		0.616	0.036	
CaO_2_ 1.86	3.170	0.010		2.63	0.27		0.700	0.020	
CPO–0.5	0.1055	0.0041		0.130	0.016		0.084	0.012	
CPO–1	0.1876	0.017		0.146	0.031		0.0908	0.0065	
CPO–1.5	0.184	0.077		0.1698	0.0033		0.111	0.053	
CPO–2	0.174	0.038	11.0	0.1254	0.0084	14.0	0.1075	0.0036	5.6
CPO–4	0.287	0.087	11.0	0.1918	0.0016	13.0	0.149	0.019	4.6
diffusion	0.0655	0.0038		0.0824	0.0088		0.044	0.013	

For the composite films, the *V*_0_ values increased with CaO_2_ content and hydrophilicity. Because CPO–4 was more hydrophilic at pH 4.5 (Figure 6), it resulted in a much higher *V*_0_ value compared to buffer pH 7.5 and D.W. The compositions CPO–2 and CPO–4 were the most efficient because they retarded the reaction, preventing the burst-release effect while enabling the highest levels of released O_2_ across different media (Table 3). The ratio (*r*) between the *V*_0_ value determined for pure CaO_2_ and the *V*_0_ values determined for CPO–2 and CPO–4 films indicated reductions in *V*_0_ of 11–14-fold in buffered media and up to 5.6-fold in D.W., likely due to the increase in pH during the kinetics in unbuffered systems. In terms of the medium, pH and ionic strength modulated the reaction kinetics, as demonstrated by changes in *V*_0_ values (Table 2) and the maximum O_2_ concentration, [O_2_], achieved across different media. For instance, in buffered systems, lower pH resulted in faster reaction rates, as evidenced by higher V_0_ values at pH 4.5 (1.92–3.17 mg L^*–*1^ h^*–*1^) compared with those at pH 7.5 (1.86–2.63 mg L^*–*1^ h^*–*1^). Furthermore, as shown in Table 3, the maximum [O_2_] achieved was also higher at pH 4.5 than at pH 7.5 for the same CaO_2_ concentration in the films. This behavior can be attributed to acidic conditions shifting the reaction equilibrium toward O_2_ production (eqs 1 and 2), whereas alkaline conditions slowed the reaction.

**Table 3:** Oxygen Concentration (mg L*^–^* ^1^) and Time (h) to Reach the Corresponding Maximum O_2_ Concentration Across Different Media

	buffer pH 4.5		buffer pH 7.5		D.W.	
	O_2_	time	O_2_	time	O_2_	time
CaO_2_ 0.936	9.63 ± 0.92	72 ± 39	14.10 ± 1.45	99 ± 1.2	8.07 ± 0.35	218 ± 17
CaO_2_ 1.86	14.60 ± 0.00	30.0 ± 5.7	17.9 ± 1.9	175 ± 37	8.65 ± 0.35	210 ± 11
CPO–0.5	7.10 ± 0.36	221 ± 20	6.45 ± 0.64	198 ± 42	6.57 ± 0.29	234.7 ± 2.9
CPO–1	6.37 ± 0.51	140 ± 78	8.83 ± 0.95	211 ± 25	7.23 ± 0.50	235.0 ± 6.0
CPO–1.5	10.1 ± 1.9	177 ± 84	9.00 ± 0.57	190 ± 18	7.5 ± 1.6	215 ± 13
CPO–2	11.4 ± 2.6	174 ± 71	14.03 ± 0.70	235.0 ± 2.6	7.167 ± 0.058	231.3 ± 2.9
CPO–4	14.8 ± 1.4	166 ± 63	12.90 ± 0.99	228 ± 14	8.00 ± 0.57	213.0 ± 7.1
diffusion	5.47 ± 0.35	233 ± 0	6.10 ± 0.30	228 ± 13	1.17 ± 0.42	74 ± 81

In general, buffered media showed higher *V*_0_ and maximum [O_2_] values than unbuffered media, which can be attributed to the pH stability provided by the buffers, allowing the reaction to proceed toward the O_2_ production. In contrast, in D. W., only at the beginning of the kinetics did the medium pH remain at 5.5 (D.W.). As the reaction proceeded, the pH increased to 11 due to Ca­(OH)_2_ formation (eq 1). This aspect inhibited the level of O_2_ production and resulted in lower *V*_0_ values.

Table 4 summarizes the literature on O_2_ release systems, including material composition, architecture, kinetic release conditions, and the maximum O_2_ concentration achieved. Compared with other 2D film architectures, CPO–4 achieved the highest maximum O_2_ concentration. Among all systems presented in Table 4, PDMS microbeads, a hydrophobic matrix (contact angle >90°),^42^ containing 50 wt % CaO_2_, achieved the highest maximum concentration (1.43 mmol L*^–^* ^1^).^43^ This result was likely due to the higher surface area of microparticles compared with films, which promotes greater oxygen release. Furthermore, compared with systems evaluated under similar pH conditions (pH 7–7.5), CPO–4 achieved O_2_ concentrations comparable to those reported for PLC particles^4^ and lignocellulose xerogels.^12^ One should notice that comparisons of maximum O_2_ concentrations across studies must account for differing evaluation timeframes. While several systems reported in Table 4 were monitored for up to 7 days to reach their maximum yields, the CPO–4 films reached equilibrium and their maximum O_2_ concentration within a 4-h window. This distinct kinetic profile characterizes the EC/CaO_2_ films as a fast-responding system, suitable for applications requiring rapid oxygenation without burst effects.

**Table 4:** Comparison of CaO_2_-Based Oxygen Release Systems Reported in the Literature

system	CaO_2_ content	architecture	*T* (^o^C)	pH	max. [O_2_] (mg L^–1^)	refs
Na–alginate	0.013 g/50 beads	beads			0.187	^44^*
PCL, pluronic	0.5–2.0% w/v	beads		7.4	0.0031	^34^
PDMS	50 wt %	microbeads	37		1.43	^43^*
CaO_2,_ PDA + lauric acid	5 mg	microparticles	RT	7	0.02	^45^*
PCL-CS	1% w/v	particles	RT	7.4	0.100	^4^
PCL-CS	1% w/v	particles	RT	7.4	0.300	^4^*
PEG, NPs	N/S	particles			0.437	^46^
CS, polaxamer 407, c–HA	0.5% w/v	hydrogel	25	7.1	0.0256	^8^
genipin-CS, catalase	0.3 wt %	hydrogel			281	^47^
CaO_2_ + PCL, PVA, CS, collagen	40 mg, 10 g gel	hydrogel	37		0.437	^48^*
PCL-CS	1% w/v	film	RT		0.100	^4^*
1,7-octadiene, urea perox.	2 mg/film	film	20	7.4	0.047	^49^
PLGA	5% w/w	film	37		0.069	^50^
collagen	0.05 mg/mL	film	24–26		0.156	^9^
quartz, stearic acid	N/S	tablet	25		0.187	^51^
HPMC-E, alkonat	31 mg/g sol.	cryogel	20	7.5	0.265	^3^
lignocellulose	69.5 wt %	xerogel	20	7.5	0.620	^12^
**CPO–4**	**62 wt %**	**film**	**20**	**4.5**	**0.460**	this work
				**7.5**	**0.400**	
				**5.5**	**0.250**	

CS: chitosan; RT: room temperature; N/S: not specified.

*No N_2_ purging.

While the CPO films reached an equilibrium within a 4-h time frame, this stabilization did not necessarily imply the complete exhaustion of the CaO_2_ source. Thus, CPO–2 and CPO–4 were selected for TGA analysis after the kinetics release to quantify the remaining CaO_2_ content (Figure S8). The CaO_2_ contents in CPO–2 and CPO–4 were 57 and 62 wt %, respectively. Comparing the initial and final CaO_2_ contents, it revealed that only 4 wt % (CPO–2) and 15 wt % (CPO–4) from the initial CaO_2_ content were consumed during the kinetics process. Thus, even though the O_2_ concentration reached an equilibrium, there was still CaO_2_ available in the films, indicating that the reaction had not fully exhausted the O_2_ source. Furthermore, TGA analyses of the composites after kinetics revealed that *T*_max_ for step I increased to 345 °C for both CPO–2 and CPO–4, approaching the *T*_max_ of pure EC (352 °C). This demonstrates that EC recovered its thermal stability when part of the CaO_2_ reacted.

Figure 8 shows the morphology of the films for compositions CPO–0.5, CPO–2, and CPO–4 after 4 h of reaction in different media. Typically, after 4 h of release, the films showed differences in color contrast, indicating that the reaction did not occur homogeneously throughout the film. Figure S14 clearly showed yellow regions corresponding to areas where CaO_2_ was still present, and white regions indicating locations where CaO_2_ had already reacted. After reaction, the surfaces of the films became more irregular after the reaction, with large pores and cracks, particularly when the maximum oxygen concentration, [O_2_], values were the highest. For example, CPO–0.5 samples showed small morphological changes (Figure 8a–d), and the maximum [O_2_] values were similar across all three media (7.1, 6.45, and 6.57 mg L^*–*1^). In contrast, SEM images for CPO–2 (Figure 8e–h) showed more pores and irregularities after kinetics in buffer pH 7.5 (Figure 8g), consistent with the highest [O_2_] value of 14.1 mg L^*–*1^. After reaction, the CPO–4 samples (Figure 8i–k) showed a clear increase of pores that likely corresponded to voids previously occupied by CaO_2_ microparticles before their reaction with water, consistent with the highest maximum [O_2_] levels achieved with CPO–4, as for instance at pH 4.5 (14.8 mg L^***–*** 1^) shown in Figure 8j. These findings suggest that increased surface porosity correlates with the reaction extent and the amount of O_2_ released.

**Figure 8:**
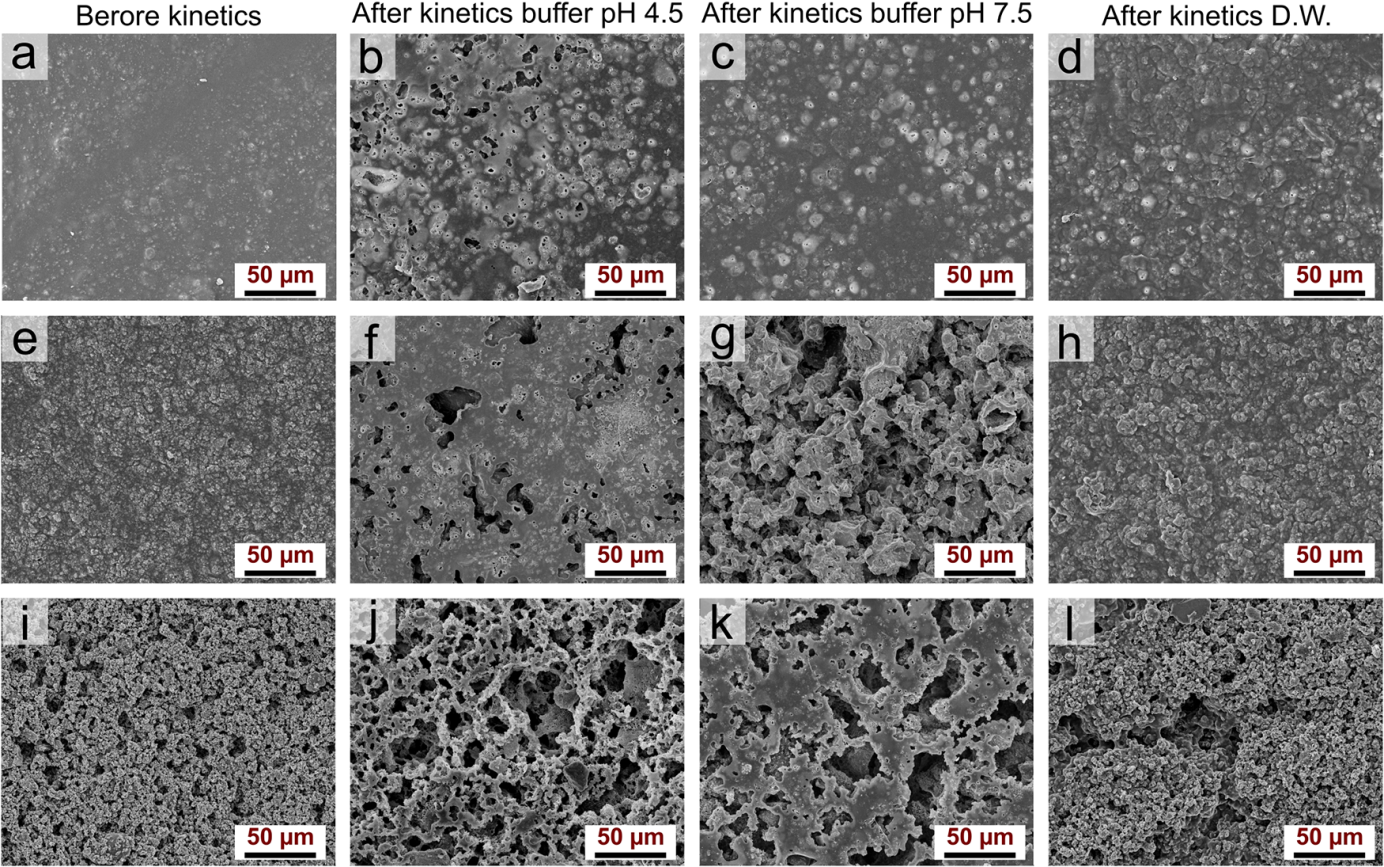
SEM images of CPO–0.5 (a) before kinetics, after kinetics in (b) buffer 4.5, (c) buffer 7.5, and (d) D.W.; CPO–2 (e) before kinetics, after kinetics in (f) buffer 4.5, (g) buffer 7.5, and (h) D.W.; CPO–4 (i) before kinetics, after kinetics in (j) buffer 4.5, (k) buffer 7.5, and (l) D.W.

### 3.5 Root Growth Analysis

As the *V*_0_ values for CPO–2 and CPO–4 differed (Table 2), while their maximum [O_2_] values were similar (Table 3), these two formulations were selected to evaluate their influence on onion root growth. For comparison, pure CaO_2_ was evaluated at the same concentration as CPO–4, along with a control group (only with the medium). The objective was to assess whether the differences in media pH and O_2_ release profiles influenced root growth.

Figures S15 and S16 show the sum of the root length and the corresponding photographs, respectively. When the medium was adjusted to buffer pH 4.5, there was no root growth (Figures S15a–c, S16a–d), whereas at buffer pH 7.5 only limited root growth was detected, with average root lengths ranging from 1 to 7.5 cm (Figures S15d–f, S16i–l). In some cases, the onion rotted completely, for example, control system at pH 7.5, where the average root length decreased over time (Figure S15d). Takakuwa et al. investigated the cytotoxic effects of acetate buffer on MH134 tumor cells to determine whether the observed toxicity resulted from acetic acid itself or from pH values below 5.0.^52^ Their results demonstrated that the cytotoxicity was primarily associated with the presence of acetic acid rather than the acidic pH. Schuerer et al. evaluated the viability of HCLE cells in the presence of different buffers, including 100 mM TRIS-HCl, which was the same concentration used in the pH 7.5 buffer.^53^ They reported that cell viability decreased to below 50% after 30 min of exposure. These studies may help explain the inhibitory effect of the buffered media on onion root growth, as acetate and Tris-HCl may exert cytotoxic effects on the roots. Therefore, subsequent root growth analyses focused on the D.W. medium, in which the influence of the O_2_-releasing materials could be more clearly observed.

To follow the O_2_ release, the pH of the D.W. medium was measured across time, as shown in Figure 9. As a control parameter, the pH of the D.W. medium without the onion was also measured to evaluate the influence of the onion on the medium pH and understand the pH changes in the system without the influence of root growth (Figure 9a). In general, with pH values ranging from 7.5 to 11, the system without the onion showed stable pH values across time. For Experiments 1 and 2, in the presence of the onion, the pH values increased and reached the maximum within 24 h, then slowly decreased with time (Figure 9a,b). After 216 h (9 days), the pH values were around 7–8.5, which would mean that the O_2_ release ceased. Another factor that could explain the pH decrease is the reaction of the Ca­(OH)_2_ with CO_2_ from the air, resulting in the formation of CaCO_3_ and H+ ions.^54^ This was also observed by Vilas Boas et al. for onion root growth systems.^12^ For Experiment 3, the pH values increased within 24 h, but then remained stable across time, because the CaO_2_ content was 5 times higher than the other two experiments (Figure 9c). After 216 h, the pH values were still around 10, indicating that the O_2_ release was still occurring.

**Figure 9:**
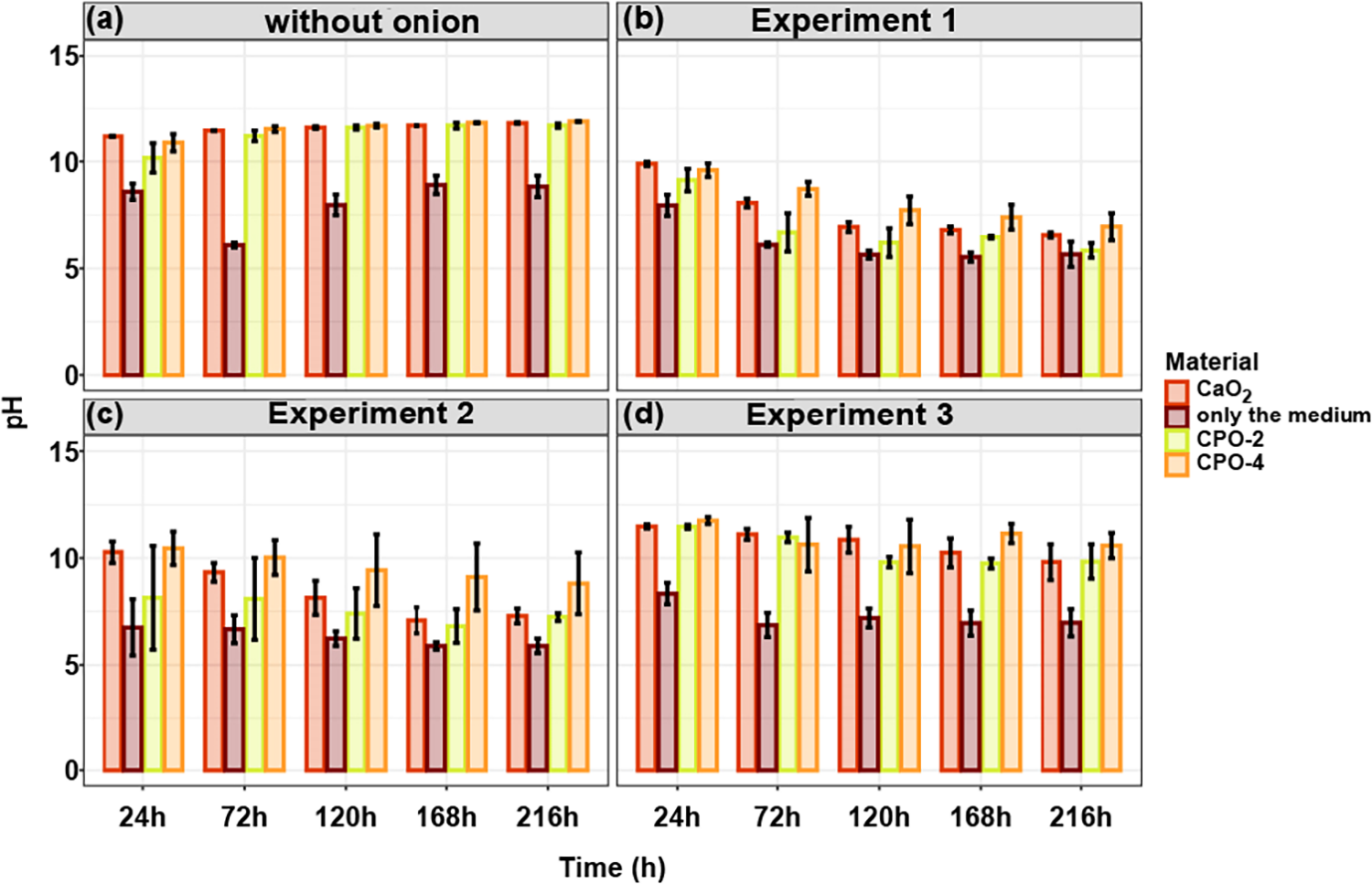
pH values for the D.W. medium for only the medium, CaO_2_, CPO–2, and CPO–4 by day (*n* = 4) (a) without onion; (b) Experiment 1; (c) Experiment 2; and (d) Experiment 3. The (a) Control represents the medium pH values measured for the systems without the onion.

Figure 10 shows the total onion root length for the D.W. medium across different experiments. To understand how each setup affected root growth, a generalized linear mixed model (GLMM) was used, with root length as the response variable. First, Experiments 1 and 2 were analyzed together to assess the impact of sample arrangement relative to sunlight. Then, Experiments 2 and 3 were combined, with the samples randomized, to evaluate how the initial CaO_2_ content influences root growth. The effect of sample position (Experiments 1 and 2) on the root growth is presented in Figure S17 and Table S18. The best model (AIC = 825), based on random-effects analysis (Table S18), showed that the variability linked to individual replicates (onion ID) was 0.43, indicating consistent results across replicates. The fixed-effects analysis found no significant difference between fixed and randomized sample positions (*p* = 0.99), suggesting that position did not affect root growth. The time factor, however, was highly significant (*p* < 0.001), indicating that root growth increased markedly over time. For treatments, there were no significant baseline differences between the control (no material) and the O_2_ sources (CaO_2_, CPO–2, and CPO–4). CPO–4 had a p-value of 0.06, near the significance threshold. The interaction between Experiment 2 and treatments revealed a slight decrease in root growth for CPO–4, while CPO–2 and CaO_2_ showed positive coefficients. None of these interactions reached statistical significance (*p* > 0.25). One should note that although the statistical analysis revealed no significant differences among the groups, Figure 10a indicated that onions exposed to the CPO–4 composite samples and positioned near the window developed the longest roots. The statistical analysis revealed no significant differences among the groups because of the presence of outliers, reflecting possible visually undetectable differences among the onions used in the study.

**Figure 10:**
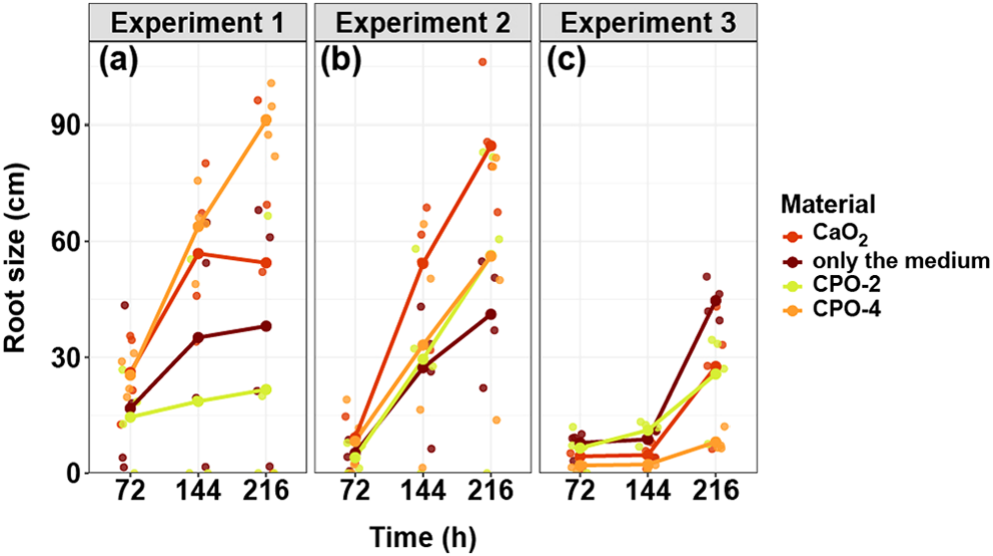
Total onion root length for D.W. medium: (a) Experiment 1; (b) Experiment 2; and (c) Experiment 3. Each jitter represents one replicate (or measure); lines represent the average value for root length for different treatments (*n* = 4).

Regarding the impact of initial CaO_2_ content (Experiments 2 vs 3), results are displayed in Figure S19 and Table S20. The chosen model (AIC = 710), with random-effects analysis, indicated that the variance among individual replicates (onion ID) was 0.29, reflecting high consistency. Fixed-effects analysis showed no significant baseline differences between treatments (*p* = 0.74) or between experiments (*p* = 0.74). The time factor remained highly significant (*p* < 0.001). Importantly, the interaction between Experiment 3 and treatment factors showed a significant decrease in root growth for CPO–4 (*p* = 0.007) and CaO_2_ (*p* = 0.023). Although the interaction between CPO–2 and Experiment 3 was not significant, it still reduced onion root growth. This indicates that higher concentrations of oxygen-releasing materials in Experiment 3 significantly inhibited root development, probably due to the cytotoxicity of different peroxide sources, including CaO_2_.^5^

In summary, the root growth analysis revealed three main findings: (i) sample positioning (fixed versus randomized) did not influence root growth; (ii) root development was strongly dependent on the growth medium, with minimal growth observed in buffered systems; and (iii) higher CaO_2_ concentrations inhibited root growth, even in D.W. systems, which might be due to high H_2_O_2_ production. These findings provide important insights into the effects of O_2_-releasing materials on root development and highlight the need to optimize CaO_2_ content to maximize oxygen-related benefits while minimizing potential phytotoxic effects.

## 4 Limitations

This study followed a single-step strategy to incorporate CaO_2_ particles in the EC matrix, aiming to retard the contact between CaO_2_ particles and water. The primary goal was fully achieved because no burst release was observed in the kinetics of the O_2_ release from the composites, regardless of the CaO_2_ loading. However, some experimental limitations were noted and might be improved in future work.

Parafilm is not a perfect sealing agent and allows for air diffusion through the medium. Ideally, the connection between the O_2_ sensor and the flask should be tailor-made, avoiding gaps for air diffusion and the use of Parafilm.

Oven drying resulted in thickness gradients and a heterogeneous distribution of CaO_2_ within the EC matrix. Hot-melt extrusion of CaO_2_ and EC into filaments may yield more homogeneous dispersions, and the resulting filaments could be pelletized, thereby enhancing the potential for large-scale production.

Another limitation refers to the onions purchased from a local market and selected solely based on visual inspection. A more rigorous assessment of their developmental stage, health status, and overall quality would be important as these factors can influence root development and may have contributed to the outliers observed in the root length measurements. Also, the onion tests would also benefit from the determination of the H_2_O_2_ concentration in order to discuss possible phytotoxic effects after long exposure.

## 5 Conclusions

This study demonstrated the potential of EC as an effective kinetic controller for the reaction between CaO_2_ and water. The hydrophobicity and surface roughness of the composite films increased with CaO_2_ content, and both characteristics contributed to mitigating the burst-release effect, promoting a slower and more sustained reaction across different media (buffered solutions at pH 4.5 and 7.5 and unbuffered distilled water).

Many systems developed for similar purposes comprise multiple components and involve multistep preparation procedures. In this work, the casting of dispersions containing CaO_2_ microparticles in ethanolic EC solutions enabled the fabrication of composite films through a simple and scalable process based on a single protective agent. This approach provides a straightforward strategy to suppress the burst release of oxygen resulting from the reaction between CaO_2_ and water while promoting a sustained oxygen release profile.

The EC/CaO_2_ composites show promise for applications requiring controlled oxygen delivery, particularly in buffered environments, such as cell culture systems. The *A. cepa* root growth assays indicated that buffered conditions combined with excess CaO_2_ can result in toxic effects. In contrast, the composite containing 62 wt % CaO_2_ in distilled water enhanced root growth, likely due to sustained oxygen release. These findings suggest that EC/CaO_2_ composites have potential for applications such as hydroponic cultivation and other systems that can benefit from a controlled oxygen supply.

## Supplementary Material



## Notes

Photographs of the CPO–4 film (a) before and (b) after cutting the edges (Figure S1), thermogravimetric analysis (TGA) decomposition events (Table S2), photograph of the experimental setup used to monitor the oxygen release as a function of time (Figure S3), statistical analysis of root size (S4), ^1^H nuclear magnetic resonance (NMR) spectrum of commercial EC (Figure S5), differential scanning calorimetry (DSC) of commercial ethyl cellulose (EC) (Figure S6), descriptive data obtained from DSC curves (Table S7), thermo-gravimetric analysis (TGA) of pure CaO_2_ and EC/CaO_2_. CPO–2 and CPO–4 formulations after kinetics release in buffer pH 7.5 (Figure S8), FTIR spectra of pure EC, pure CaO_2_, and the EC/CaO_2_ films (Figure S9), surface profilometry (CCI) for EC and EC/CaO_2_ films over 300 μm × 300 μm areas (Figure S10), surface profilometry (CCI) for CPO–2 and CPO–4 films from the center to the edge of the films over 300 μm × 300 μm areas (Figure S11), contact angle measurements as a function of time (Figure S12), SEM images of EC films after stability tested at different media (Figure S13), photographs of both sides (a and b) of a CPO–2 film after 4 h release in a pH 4.5 buffer (Figure S14), sum of the root length (Figure S15) and the corresponding photographs (Figure S16), the effect of sample position (Experiments 1 and 2) on the root growth (Figure S17 and Table S18), the impact of initial CaO_2_ content (Experiments 2 and 3) on the root growth (Figure S19 and Table S20) (PDF)
