# Supplementary material for: Oxygen Delivery from Ethylcellulose/Calcium Peroxide Composite Films: Effects of Composition and Medium pH
Source: ACS Omega. 2026 Jul 14;11(29):44480–92. doi: 10.1021/acsomega.6c05828 (PMC13425337; doi:10.1021/acsomega.6c05828)
Supplement: Supplementary file 1 [file ao6c05828_si_001.pdf]

Oxygen delivery from ethylcellulose/calcium peroxide composite films: Effects of composition and medium pH

Camila Gruber Chiaregato and Denise Freitas Siqueira Petri*

Instituto de Química, Universidade de São Paulo, 05508-900, São Paulo, Brazil.

E-mail: *dfsp@iq.usp.br

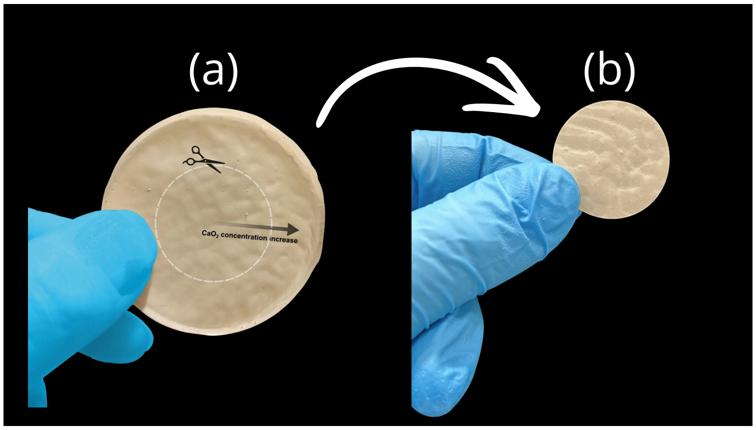

Figure S1: Photographs of the CPO-4 film (a) before and (b) after cutting the edges.

Table S2: Thermogravimetric analysis (TGA) decomposition events.

			CPO-2		CPO-4	
**Event**	**EC**	**CaO_2**	**Initial**	**Post-kinetics**	**Initial**	**Post-kinetics**
**Moisture** (Δ %)	3.04	2.78	0.16	0.71	1.53	0.64
**Stage I**						
Mass loss (Δ %)	93.8	–	30.21	26.59	17.07	30.7
T_{max} (°C)	352	–	332	345	325	345
**Stage II**						
Mass loss (Δ %)	–	19.69	13.80	11.81	14.28	10.52
T_{max} (°C)	–	381	387	394	383	394
**Stage III**						
Mass loss (Δ %)	–	3.91	6.68	7.95	7.50	8.63
T_{max} (°C)	–	611	595	618	600	617
**Ashes** (%)	3.16	73.62	49.15	52.92	58.92	49.51

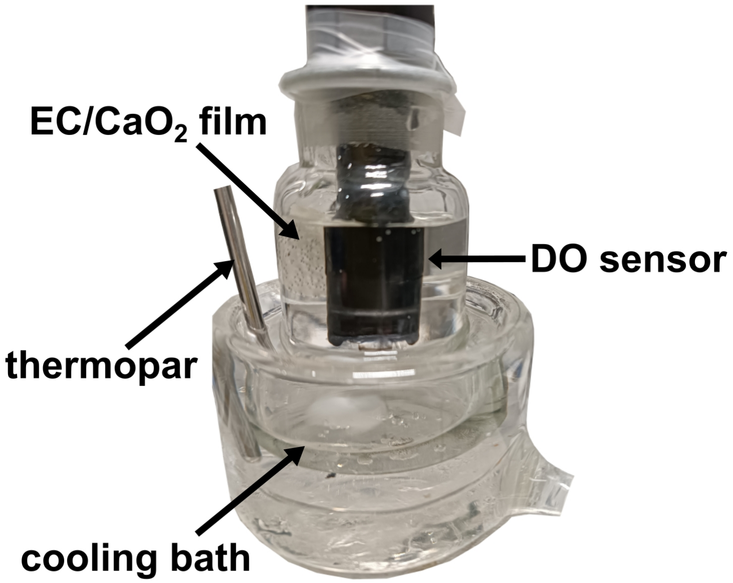

Figure S3: Photograph of the experimental setup used to monitor the oxygen release as a function of time.

S4 Statistical analysis of root size

For the root growth analysis, Generalized Linear Mixed Models (GLMMs) were structured to evaluate the effects of the treatment (*Material*), *Time*, and *Experiment*.[1–3] Root size was analyzed as a continuous positive variable and modeled using a Tweedie distribution with a log link. Because the experimental comparisons required different analytical assumptions, two statistical strategies were employed:

1. **Additive Model (Experiments I vs. II):** To compare the first two experiments, an interaction model was used. The model included *Material*, *Time*, and *Experiment* as fixed effects, and *Onion ID* as a random intercept:

$$\text{root\_size} \sim \text{Material} + \text{Time} + \text{Exp} + (1 \mid \text{Onion\_ID}) \tag{S1}$$

2. **Interaction Model (Experiments II vs. III):** To compare the second and third experiments, an interaction model was employed to assess whether the materials' performance differed depending on the experiment. This model included the interaction term between *Material* and *Experiment*:

$$\text{root\_size} \sim \text{Material} \times \text{Exp} + \text{Time} + (1 \mid \text{Onion\_ID}) \tag{S2}$$

The general equation for the predicted mean root size, μ_{ijkl}, for material i, experiment j, at time k, for the individual onion l (incorporating all potential terms from the interaction model) followed:

$$\log(\mu_{ijkl}) = \beta_0 + \sum_{i=1}^{M-1} \beta_{M_i} \cdot \text{Material}_i + \beta_T \cdot \text{Time}_k + \beta_{E_j} \cdot \text{Exp}_j + \sum_{i=1}^{M-1} \beta_{I_{ij}} \cdot (\text{Material}_i \times \text{Exp}_j) + u_l \tag{S3}$$

Where:

- β_0 is the fixed intercept of the model;
- β_{M_i} are the fixed-effect coefficients associated with the $M - 1$ levels of the factor *Material*, based on reference (dummy) coding;
- β_T is the fixed-effect coefficient for the continuous covariate *Time*;
- β_{E_j} is the fixed-effect coefficient for the *Experiment* level;
- $\beta_{I_{ij}}$ represents the interaction coefficients between *Material* and *Experiment* (evaluated in the II vs. III model);
- $u_l \sim \mathcal{N}(0, \sigma^2_{\text{onion\_id}})$ represents the random effect associated with the grouping factor *Onion ID*, accounting for repeated measures within the same individual onion.

The response variable Y_{ijkl} is assumed to follow a distribution from the Tweedie family, characterized by a mean parameter μ_{ijkl}, a dispersion parameter ϕ, and a power index p, such that:

$$Y_{ijkl} \sim \text{Tweedie}(\mu_{ijkl}, \phi, p), \text{ with } \phi > 0, 1 < p < 2 \tag{S4}$$

This specification allows the modeling of continuous positive data with positive skewness and potential excess zeros. To understand the effects of different treatments and their interactions on root growth, the exponentiated coefficients ($\exp(\beta)$) were calculated for each model term. These exponentiated values represent the multiplicative effect (percentage change) in root size relative to the reference categories.

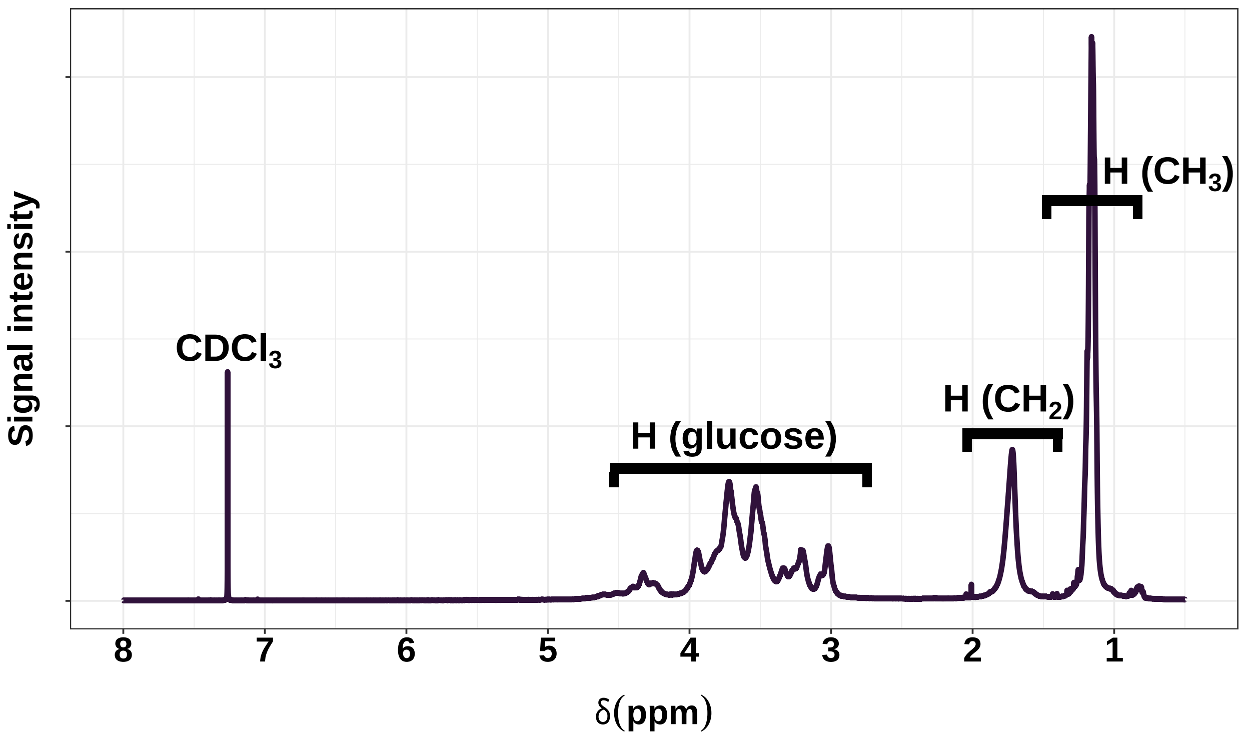

Figure S5: ^{1}H Nuclear magnetic resonance (NMR) spectrum of commercial EC.

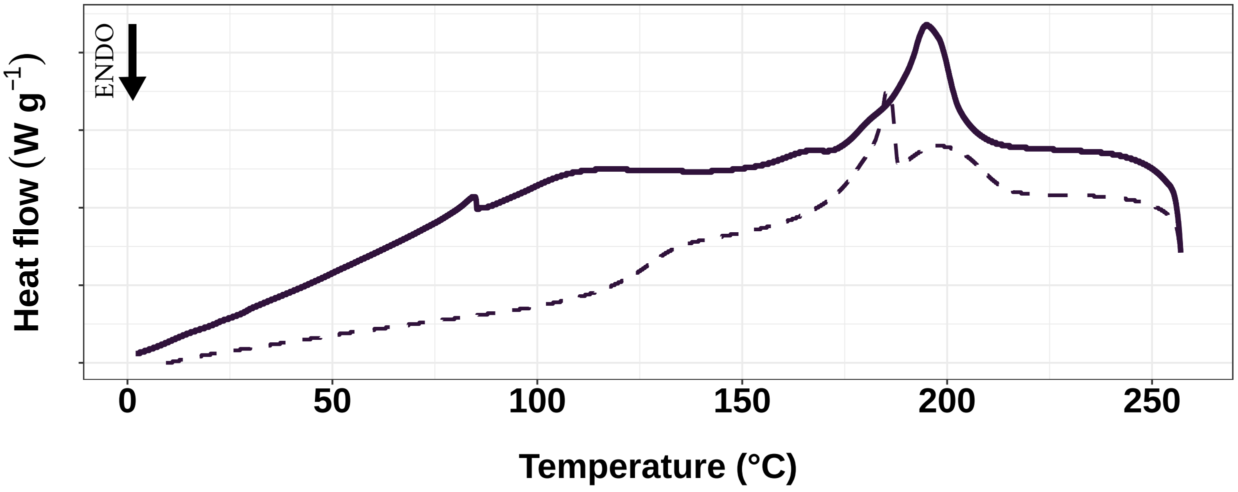

(a) Cooling curve.

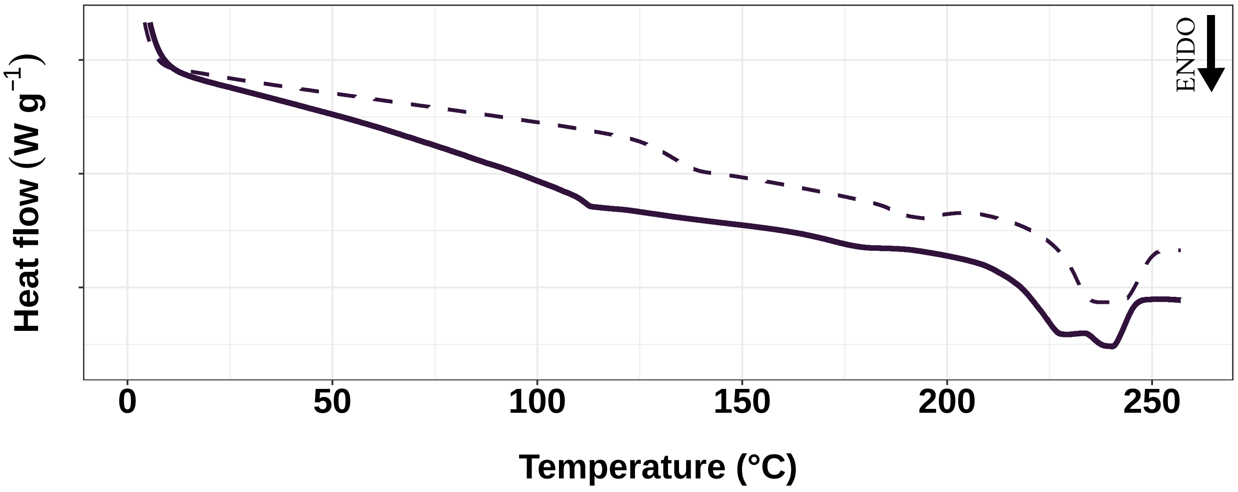

(b) 2nd heating curve.

Figure S6: Differential scanning calorimetry (DSC) of commercial ethyl cellulose (EC) (endothermic events down). Dash-line: pin-holed pans. Line: sealed pan.

Table S7: Descriptive data obtained from DSC curves. *1st crystallization peak; ** 2nd crystallization peak; † 1st fusion peak; †† 2nd fusion peak.

Type of pan	T_g (°C)	$\Delta H_{crys.}$ (J/g)	$T_{crys.}$ (°C)	ΔH_{fusion} (J/g)	T_{fusion} (°C)
Pin-holed	132	9.31	185*<br>198**	9.57	236
Sealed	111	7.09	195	10.26	227†<br>238††

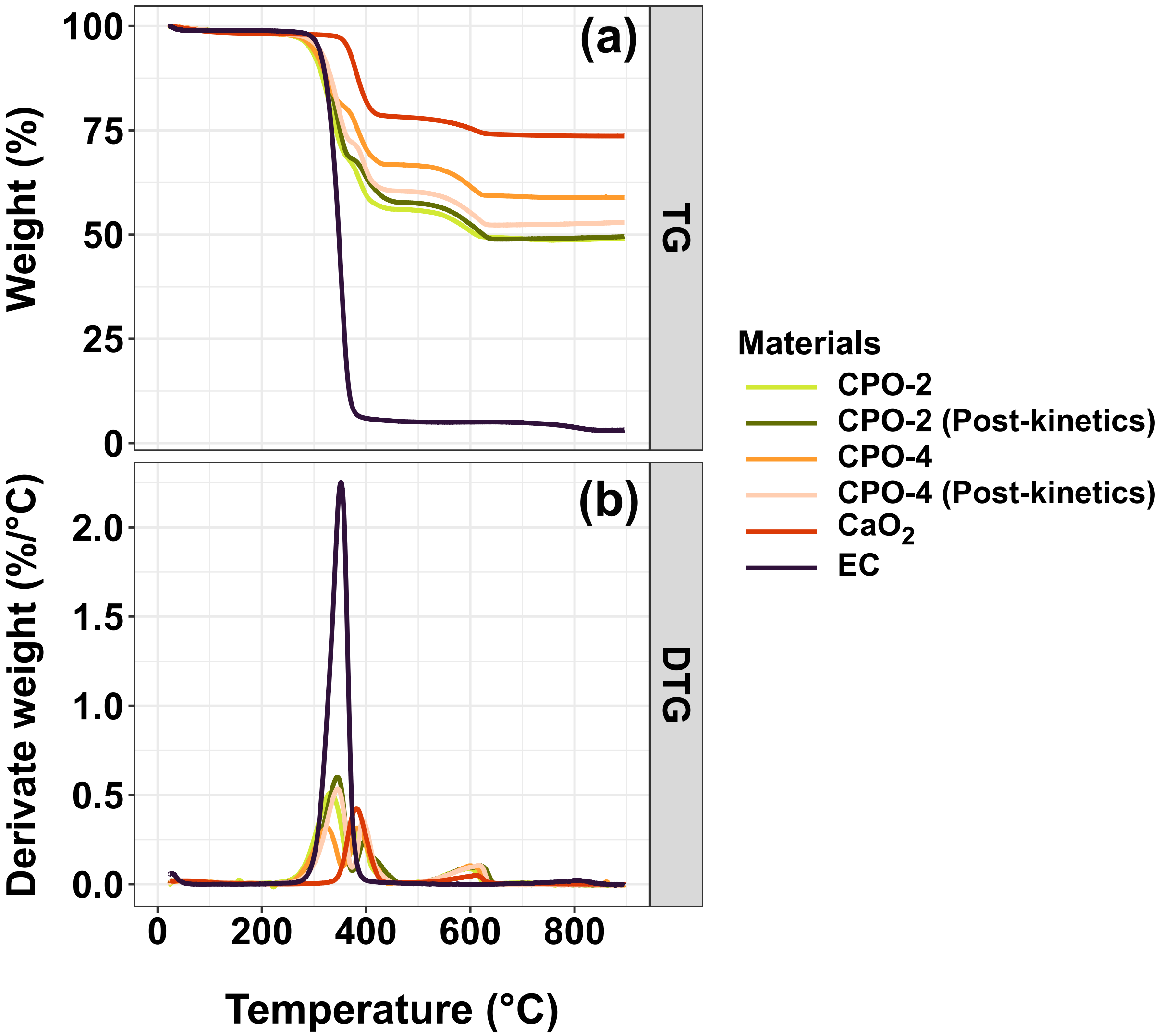

Figure S8: Thermogravimetric analysis (TGA) of pure CaO_2 and EC/CaO_2. CPO-2 and CPO-4 formulations were analyzed after kinetics release in buffer pH 7.5.

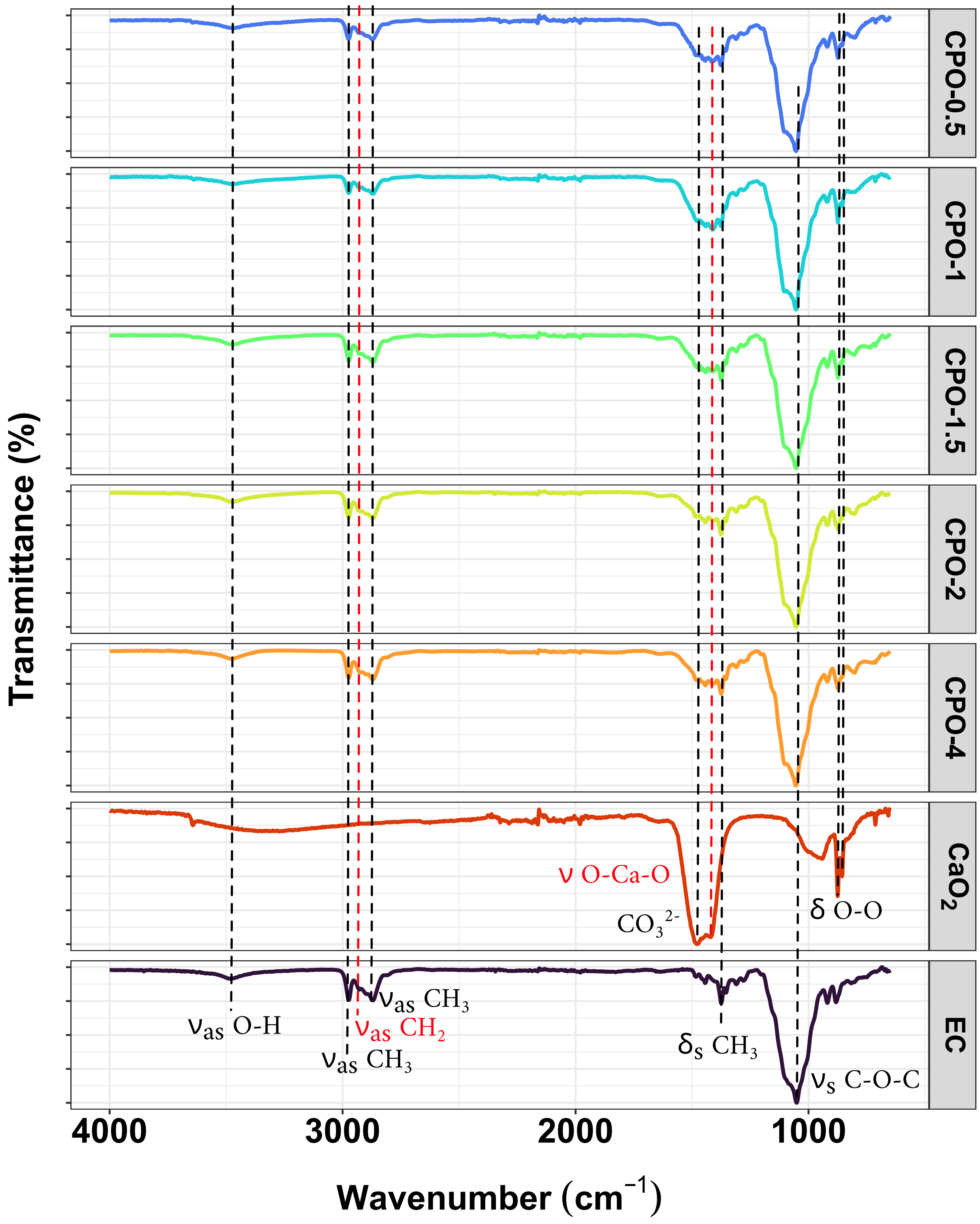

Figure S9: Fourier-transform infrared spectroscopy (FTIR) spectra of pure materials (EC and CaO_2) and EC/CaO_2 films.

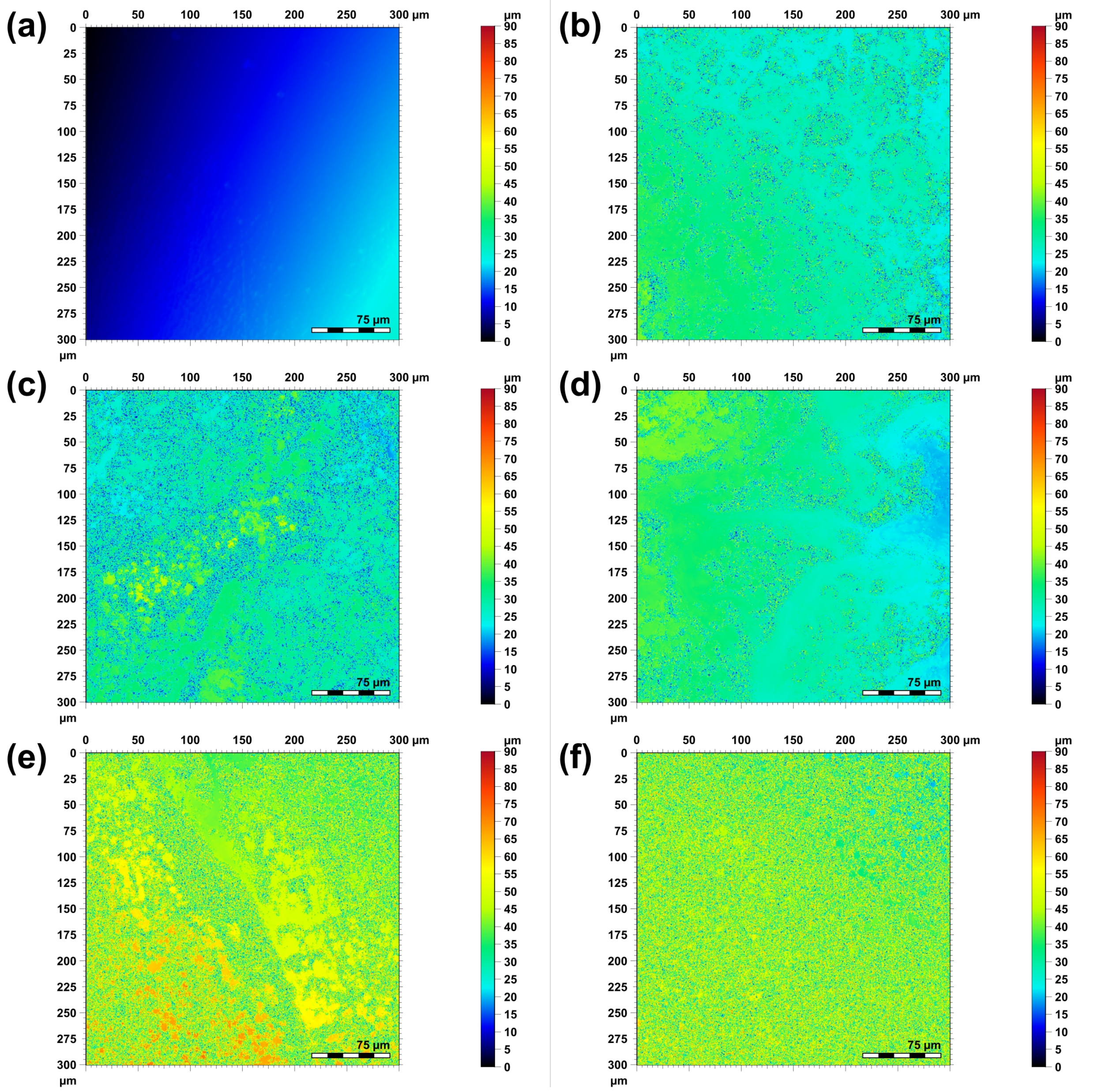

Figure S10: Surface profilometry (CCI) for films: (a) CPO-0; (b) CPO-0.5; (c) CPO-1; (d) CPO-1.5; (e) CPO-2; (f) CPO-4.

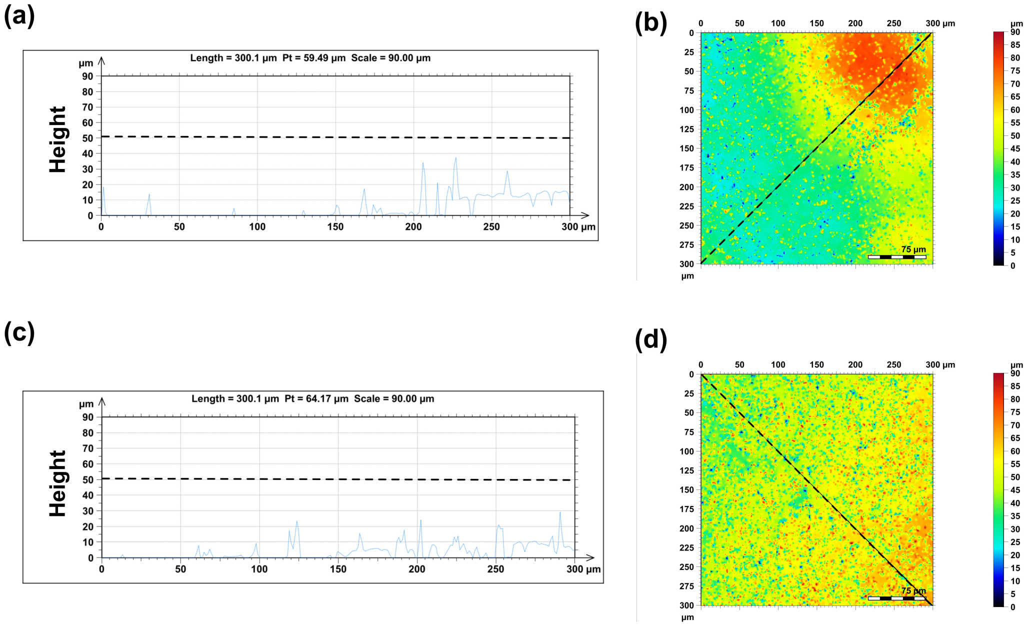

Figure S11: Surface profilometry (CCI) for **CPO-2** (a and b) and **CPO-4** (c and d) films from the center to the edge of the films over 300 μm x 300 μm areas: (a and c) Height profile across the surface cross-section; and (b and d) Contour plots of surface textures.

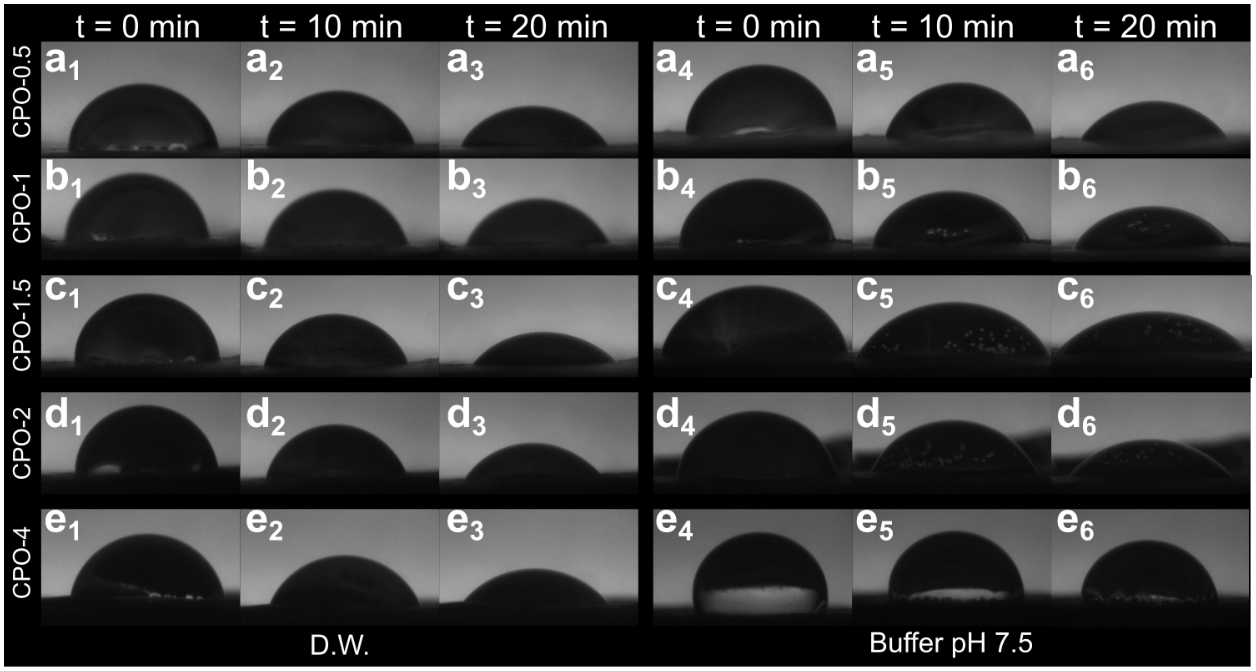

Figure S12: Contact angle measurement photographs using the sessile drop methodology for: CPO-0.5 and **D.W. medium** (a_1) 0 min; (a_2) 10 min; (a_3) 20 min; **buffer pH 7.5** (a_4) 0 min; (a_5) 10 min; (a_6) 20 min; CPO-1 and **D.W. medium** (b_1) 0 min; (b_2) 10 min; (b_3) 20 min; **buffer pH 7.5** (b_4) 0 min; (b_5) 10 min; (b_6) 20 min; CPO-1.5 and **D.W. medium** (c_1) 0 min; (c_2) 10 min; (c_3) 20 min; **buffer pH 7.5** (c_4) 0 min; (c_5) 10 min; (c_6) 20 min; CPO-1 and **D.W. medium** (d_1) 0 min; (d_2) 10 min; (d_3) 20 min; **buffer pH 7.5** (d_4) 0 min; (d_5) 10 min; (d_6) 20 min; CPO-4 and **D.W. medium** (e_1) 0 min; (e_2) 10 min; (e_3) 20 min; **buffer pH 7.5** (e_4) 0 min; (e_5) 10 min; and (e_6) 20 min.

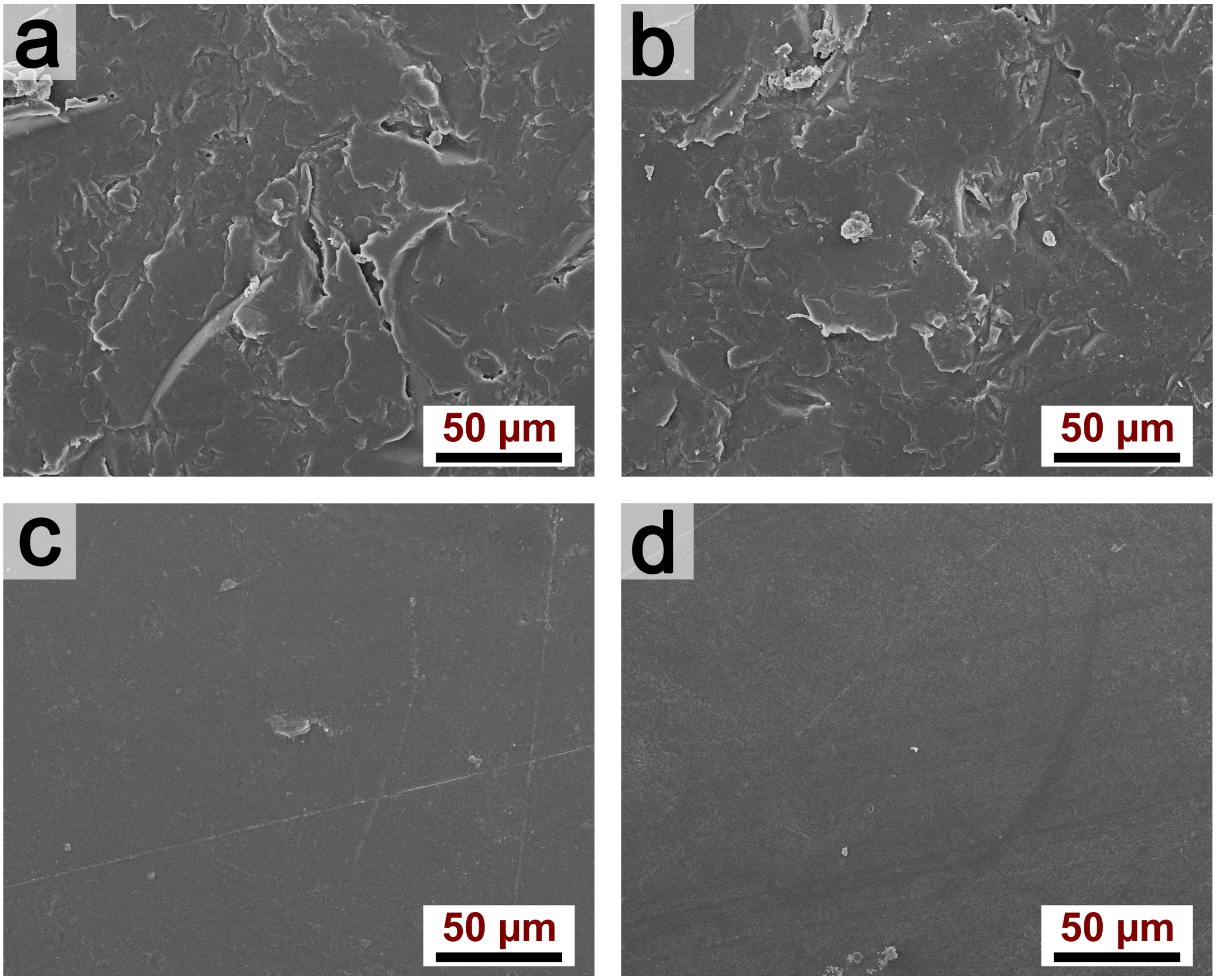

Figure S13: SEM images of EC films after stability test in (a) acetic acid-acetate buffer, pH 4.5; (b) D.W., pH 5.5; (c) Tris-HCl buffer, pH 7.5; (d) NaOH solution, pH 11.

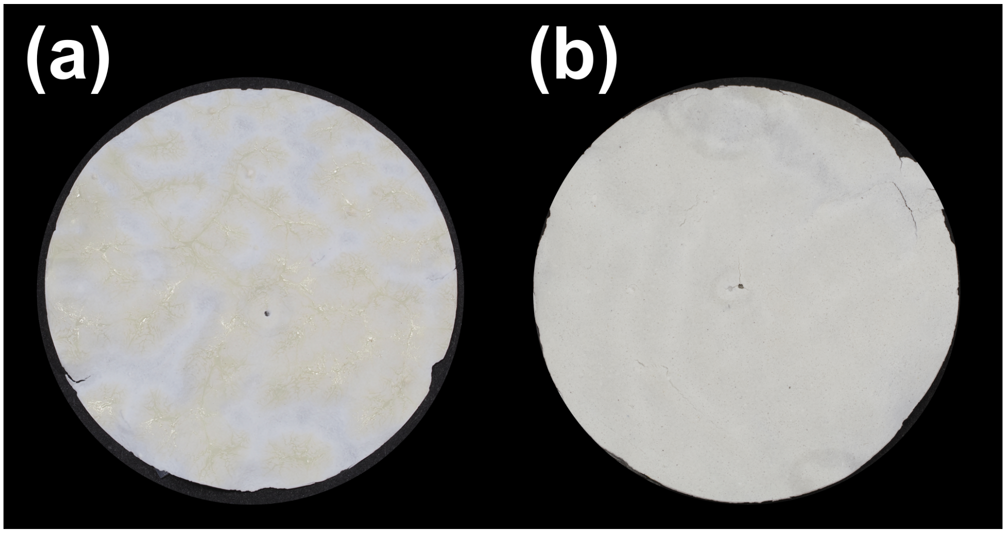

Figure S14: Photographs of both sides (a and b) of a CPO-2 film after 4 h release in pH 4.5 buffer

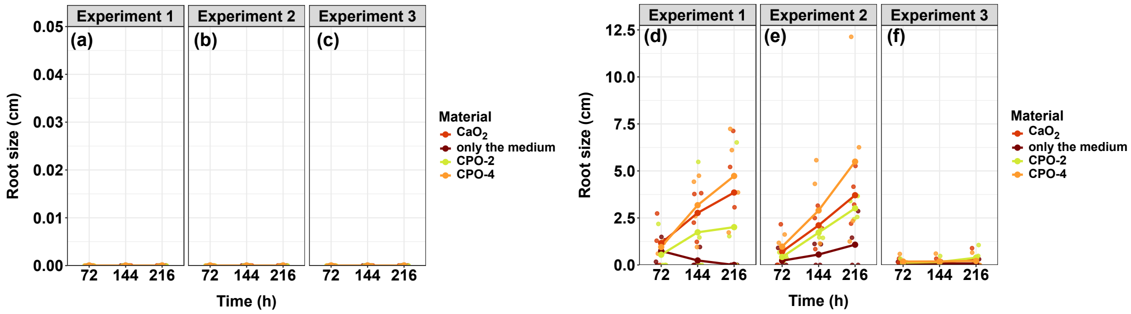

Figure S15: Onion root length for **Buffer pH 4.5 medium**: (a) Experiment 1; (b) Experiment 2; (c) Experiment 3; **Buffer pH 7.5 medium**: (d) Experiment 1; (e) Experiment 2; and (f) Experiment 3. Each jitter represents one replicate (or measure); line represent the average value for root length for different treatment.

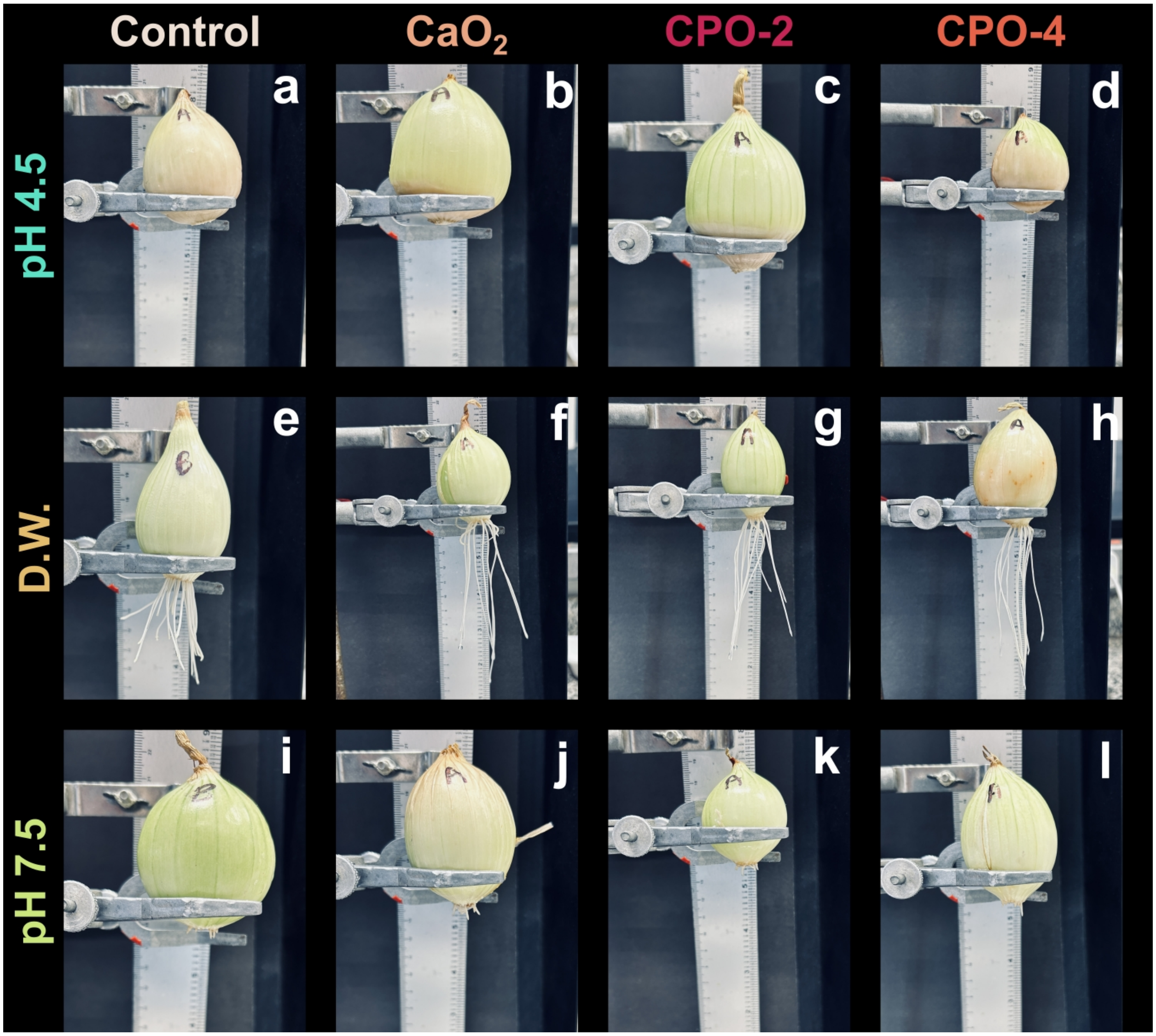

Figure S16: Photographs of onion roots length after 216 h for Experiment II: **buffer pH 4.5 medium** (a) Control; (b) CaO_2; (c) CPO-2; (d) CPO-4; **D.W. medium** (e) Control; (f) CaO_2; (g) CPO-2; (h) CPO-4, **buffer pH 7.5 medium** (i) Control; (j) CaO_2; (k) CPO-2; and (l) CPO-4.

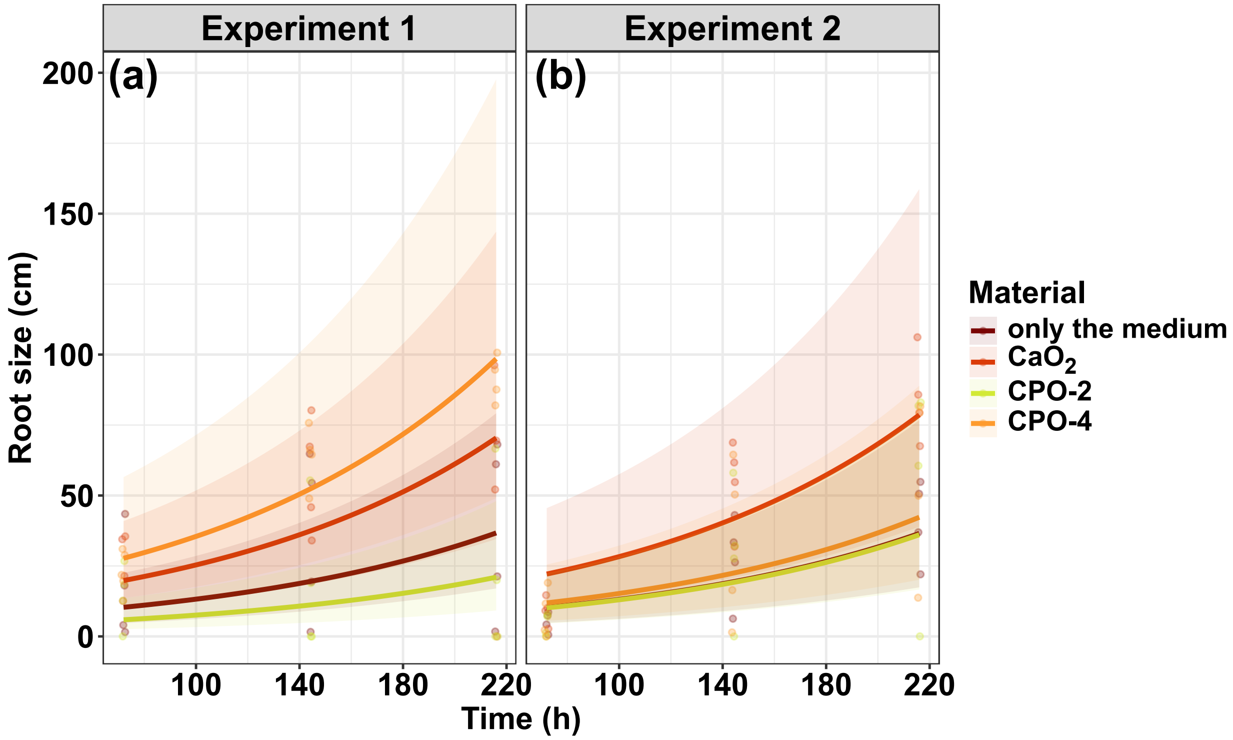

Figure S17: Predicted onion root length calculated by comparing Experiments 1 vs 2 with GLMM analysis with experimental values for D.W. medium.

Table S18: Summary of the GLMM (Tweedie family with log link) comparing Experiment 1 vs 2. The table presents the fixed effects coefficients ($\hat{\beta}$), standard errors (SE), 95% confidence intervals (CI), and exponentiated values ($\exp(\hat{\beta})$), representing the multiplicative effect on root size. Variance and standard deviation are reported for the random effect (Onion ID). $^{\cdot}p < 0.1$; $^{*}p < 0.05$; $^{**}p < 0.01$; $^{***}p < 0.001$.

Fixed effects

**Parameter**	$\hat{\beta}$	**SE**	**95% CI**	**z-value**	**p-value**	**exp($\hat{\beta}$)**	**exp(95% CI)**
Intercept	1.70	0.42	0.88 – 2.53	4.052	$<0.001^{***}$	5.48	2.41 – 12.50
Calcium peroxide	0.65	0.53	-0.38 – 1.68	1.236	0.216	1.92	0.68 – 5.39
CPO-2	-0.56	0.56	-1.65 – 0.53	-1.006	0.314	0.57	0.19 – 1.70
CPO-4	0.99	0.52	-0.04 – 2.01	1.884	$0.060^{\cdot}$	2.68	0.96 – 7.49
Experiment 2	-0.005	0.54	-1.06 – 1.05	-0.009	0.993	1.00	0.35 – 2.86
Time	0.0088	0.0010	0.007 – 0.011	8.864	$<0.001^{***}$	1.01	1.01 – 1.01
Calcium peroxide : Exp 2	0.11	0.74	-1.33 – 1.56	0.156	0.876	1.12	0.27 – 4.75
CPO-2 : Exp 2	0.55	0.77	-0.97 – 2.06	0.707	0.480	1.73	0.38 – 7.84
CPO-4 : Exp 2	-0.84	0.75	-2.31 – 0.63	-1.123	0.261	0.43	0.10 – 1.87

Random effects

**Group**	**Parameter**	**Variance**	**Std. Dev.**
Onion ID	Intercept	0.43	0.66

Model equation: Root size $\sim$ Material $\times$ Exp + Time + (1 | Onion ID)

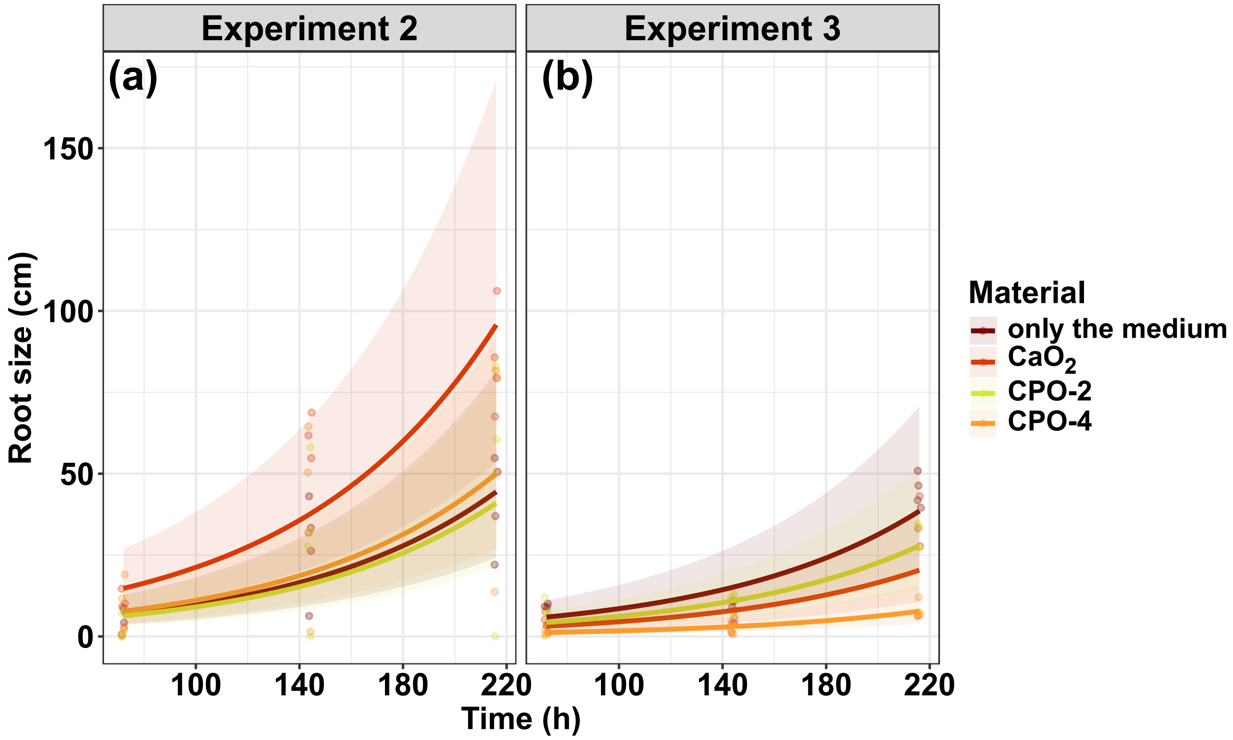

Figure S19: Predicted onion root length calculated by comparing Experiments 2 vs 3 with GLMM analysis with experimental values for D.W. medium.

Table S20: Summary of the GLMM (Tweedie family with log link) comparing Experiment 2 vs 3. The table presents the fixed effects coefficients ($\hat{\beta}$), standard errors (SE), 95% confidence intervals (CI), and exponentiated values ($\exp(\hat{\beta})$), representing the multiplicative effect on root size. The interaction terms evaluate how materials perform differently in Experiment 3 compared to Experiment 2. Variance and standard deviation are reported for the random effect (Onion ID). *p $<$ 0.05; **p $<$ 0.01; ***p $<$ 0.001.

Fixed effects

**Parameter**	$\hat{\beta}$	**SE**	**95% CI**	**z-value**	**p-value**	**exp($\hat{\beta}$)**	**exp(95% CI)**
Intercept	0.99	0.35	0.31 – 1.67	2.852	0.004**	2.69	1.36 – 5.30
Calcium peroxide	0.77	0.42	-0.06 – 1.60	1.809	0.070	2.15	0.94 – 4.95
CPO-2	-0.08	0.45	-0.96 – 0.80	-0.181	0.857	0.92	0.38 – 2.22
CPO-4	0.12	0.43	-0.73 – 0.97	0.272	0.785	1.13	0.48 – 2.64
Experiment 3	-0.14	0.43	-1.00 – 0.71	-0.330	0.742	0.87	0.37 – 2.03
Time	0.013	0.0009	0.011 – 0.015	13.674	<0.001***	1.01	1.01 – 1.01
Calcium peroxide × Exp 3	-1.40	0.62	-2.62 – -0.19	-2.271	0.023*	0.25	0.07 – 0.82
CPO-2 × Exp 3	-0.24	0.63	-1.47 – 0.99	-0.386	0.699	0.78	0.23 – 2.69
CPO-4 × Exp 3	-1.72	0.64	-2.98 – -0.46	-2.682	0.007**	0.18	0.05 – 0.63

Random effects

**Group**	**Parameter**	**Variance**	**Std. Dev.**
Onion ID	Intercept	0.29	0.54

Model equation: Root size $\sim$ Material $\times$ Exp + Time + (1 | Onion ID)

References

(1) McGillycuddy, M.; Popovic, G.; Bolker, B. M.; Warton, D. I. Parsimoniously Fitting Large Multivariate Random Effects in glmmTMB. *Journal of Statistical Software* **2025**, *112*, 1–19.

(2) Brooks, M. E.; Kristensen, K.; van Benthem, K. J.; Magnusson, A.; Berg, C. W.; Nielsen, A.; Skaug, H. J.; Mächler, M.; Bolker, B. M. glmmTMB Balances Speed and Flexibility Among Packages for Zero-inflated Generalized Linear Mixed Modeling. *The R Journal* **2017**, *9*, 378–400.

(3) Bates, D.; Mächler, M.; Bolker, B.; Walker, S. Fitting Linear Mixed-Effects Models Using Lme4. *Journal of Statistical Software* **2015**, *67*, 1–48.
